# Comparative transcriptomics of anthocyanin accumulation in the pericarp of pigmented purple corn

**DOI:** 10.1007/s00122-025-05137-x

**Published:** 2026-01-17

**Authors:** Anderson A. Holly, Paulsmeyer N. Michael, Juvik A. John

**Affiliations:** 1 Department of Crop Sciences, University of Illinois, Urbana-Champaign, Urbana, IL 61801 USA; 2 Vegetable Crops Research Unit, USDA-ARS, University of Wisconsin, Madison, WI 53706 USA

## Abstract

**Key message:**

RNA-seq, bulked segregant analysis, anthocyanin quantification, and WGCNA identified Pl1, Lc1, P1, and Wrky33 as important regulatory factors for enhancing anthocyanin content in maize pericarp.

**Abstract:**

Anthocyanins are plant pigments that can be used as natural food colorants. We developed Midwestern, purple corn lines with enhanced anthocyanin content exclusively in the pericarp tissue layer: two lines near-isogenic to elite inbreds B73 and Mo17 (B73 Color Converted and Mo17 Color Converted, respectively) and two recombinant inbred lines (RILs) with diverse anthocyanin profiles (Amazonas and Maize Morado). In Experiment 1, a time-series, RNA-sequencing (RNA-seq) analysis of whole pericarp tissue was conducted on three pigmented genotypes (B73 Color Converted, Amazonas, and Maize Morado). Ultra-High-Pressure Liquid Chromatography (UHPLC) identified a dramatic increase in anthocyanin accumulation between 15 and 20 days after pollination (DAP) in pigmented genotypes. Bulk segregant analysis discovered *Leaf color1* (*Lc1*) and *Purple plant1* (*Pl1*) as the major contributors to pericarp pigmentation in B73 Color Converted. Additional loci *Bronze2* (*Bz2*) and *Pericarp color1* (*P1*) were also donated by the purple parent. In Experiment 2, RNA-seq was performed on 18 DAP kernels of four pigmented maize lines (B73 Color Converted, Mo17 Color Converted, Amazonas, and Maize Morado), comparing pigmented and unpigmented pericarp fractions from bulked individual kernels. Upregulation of *Lc1*, *Pl1*, and *P1* suggests a distinct MBW protein complex in pigmented pericarp. Correlational analyses of 18 DAP pigmented pericarp fractions revealed enriched expression of anthocyanin transporters, *Bz2* and *Multidrug resistance-associated protein3* (*Mrpa3*), and a candidate transcription factor, *WRKY-transcription factor 33* (*Wrky33*). These candidate genes can be used in breeding programs as a source of natural food and beverage colorants and improve our understanding of the mechanisms underlying maize pericarp pigmentation.

**Supplementary Information:**

The online version contains supplementary material available at https://doi.org/10.1007/s00122-025-05137-x.

## Introduction

Anthocyanins are pigments found throughout plant tissues. As antioxidative compounds, they function in a protective role against stressors, specifically oxidative stress induced by biotic and abiotic factors, such as pathogens and drought, respectively (Petroni et al. 2014; Dong and Lin 2021; Naing and Kim 2021). Anthocyanins are a class of flavonoids housed within the broader phenylpropanoid biosynthetic pathway. They are derived from anthocyanidins, a diverse family of compounds differentiated by the position of hydroxylation and methoxylation on their aromatic, B-ring (Valls et al. 2009; Riaz et al. 2016; Sendri and Bhandari 2024).

Maize accumulates three specific anthocyanidins: cyanidin, peonidin, and pelargonidin (Paulsmeyer et al. 2017). Once glycosylated, anthocyanidins become anthocyanins and may undergo additional modifications, including acylation, condensation with flavanols, and non-covalent copigmentation with other flavonoids, to influence hue and color stability (Asen et al. 1972; Riaz et al. 2016; Zhao et al. 2021). Anthocyanins are stored within the vacuole, and their hue, composition, and stability are largely influenced by the vacuolar pH, with visible red pigmentation present at low pHs (pH ≤ 3) where the flavylium cation prevails (Zhao et al. 2021).

With increased public demand for less processed foods, natural pigments are of interest to the food industry as an affordable and healthier alternative to synthetic food dyes. Relative to other natural pigments, anthocyanins are water soluble, facilitating simpler extractions without the use of harsh chemicals. Additionally, anthocyanin consumption is associated with health-promoting benefits such as lower risks of cardiovascular disease, cancer, obesity, and inflammation (Khoo et al. 2017). Optimization of anthocyanin production in a staple crop, such as maize, can seamlessly integrate natural colorants into a pre-existing, multi-billion-dollar supplement market (United States Department of Agriculture 2024). We initiated an anthocyanin-rich, pericarp-pigmented maize breeding program to address the need for alternative sources of natural dyes for processed foods and beverages. Efforts were focused on optimizing anthocyanin content in maize kernels as vegetables that comply with FDA requirements for “fruit or vegetable use for color” (CFR-Code of Federal Regulations Title 21). Relative to other perishable, anthocyanin-rich crops, maize was specifically selected for its unique dry-down ability, allowing for long-term storage prior to pigment extraction (Chatham et al. 2020). Anthocyanin biosynthesis has been well detailed in the maize aleurone layer, the kernel cell layer surrounding the endosperm, though the pericarp layer, the outermost layer of the kernel, reportedly accumulates higher quantities of anthocyanins that are more extractable (Sharma et al. 2011; Paulsmeyer et al. 2017; Chatham and Juvik 2021). While it is assumed that anthocyanin biosynthesis is similar between maize aleurone and pericarp tissues, each tissue has a distinct genetic origin, with the diploid pericarp derived from the maternal ovary and the triploid aleurone derived from the double fertilization of the ovule (García-Lara et al. 2019; Paulsmeyer and Juvik 2023a). Breeding efforts focused on pericarp pigmentation can produce larger quantities of anthocyanins to more efficiently meet demands for natural colorants.

Transcriptional regulation of anthocyanin biosynthesis is highly conserved among higher plants and facilitated by regulatory gene products. Activation of anthocyanin biosynthetic genes requires the assembly of the MBW (R2R3-**M**YB, **b**HLH, and **W**D-repeat proteins) complex. Pericarp pigmentation is assumed to be under similar regulatory mechanisms as vegetative tissues (Chatham and Juvik 2021). Based on this report, the predicted R2R3-MYB (myeloblastosis protein) of the MBW complex is *Purple plant 1* (*Pl1*). Multiple bHLH (basic helix-loop-helix) proteins exist in maize—either the *Leaf color1* (*Lc1*) gene within the *Colored1* (*R1*) locus or the *B1* (*Booster1*) gene confers pericarp pigmentation (Ludwig et al. 1989; Ludwig and Wessler 1990). The WD40 (WD-repeat) domain containing protein in pericarp is unknown. In aleurone, *Pale aleurone color1* (*Pac1*) encodes the WD40 protein, but is not predicted to function in the pericarp MBW complex (Chatham & Juvik 2021; Selinger & Chandler 1999; Paulsmeyer and Juvik 2023b).

Although the molecular mechanisms of anthocyanin biosynthesis in maize aleurone are well characterized, few studies have conducted comprehensive transcriptome analyses of pericarp-pigmented maize tissues across development. To our knowledge, none have focused on near-isogenic or recombinant inbred anthocyanin-rich lines adapted to the Midwest. While a previous study aligned transcriptomic data from two pericarp-pigmented lines against the B73 reference genome (Li et al. 2022), we provide a more appropriate alignment using the transcriptome of a pericarp-pigmented line near-isogenic to B73. Additionally, transcriptomic analysis was conducted on a pericarp-pigmented line near-isogenic to Mo17 and two recombinant inbred lines (RILs) exhibiting high yields of diverse anthocyanins to identify novel candidate genes.

The aim of our study was to analyze developmental trends in gene expression of near-isogenic and inbred pericarp-pigmented maize lines to identify candidate genes for anthocyanin accumulation. Conservation of anthocyanin biosynthetic processes across kernel tissues of unique genetic origins (maternal and embryonic) was assessed by comparing the maize pericarp layer to the well-characterized maize aleurone layer. Here, we conducted two RNA-seq experiments: 1) using three pigmented lines compared to an unpigmented B73 control at three timepoints [10, 15, and 20 days after pollination (DAP)] and 2) using pigmented and unpigmented pericarp tissue fractions from the same kernel harvested at a timepoint of major anthocyanin accumulation in the pericarp (18 DAP). Understanding the temporal expression of anthocyanin biosynthetic genes during pericarp development is important for optimizing anthocyanin content through breeding programs for natural food colorants.

## Materials and methods

### Plant materials

**Fig. 1:**
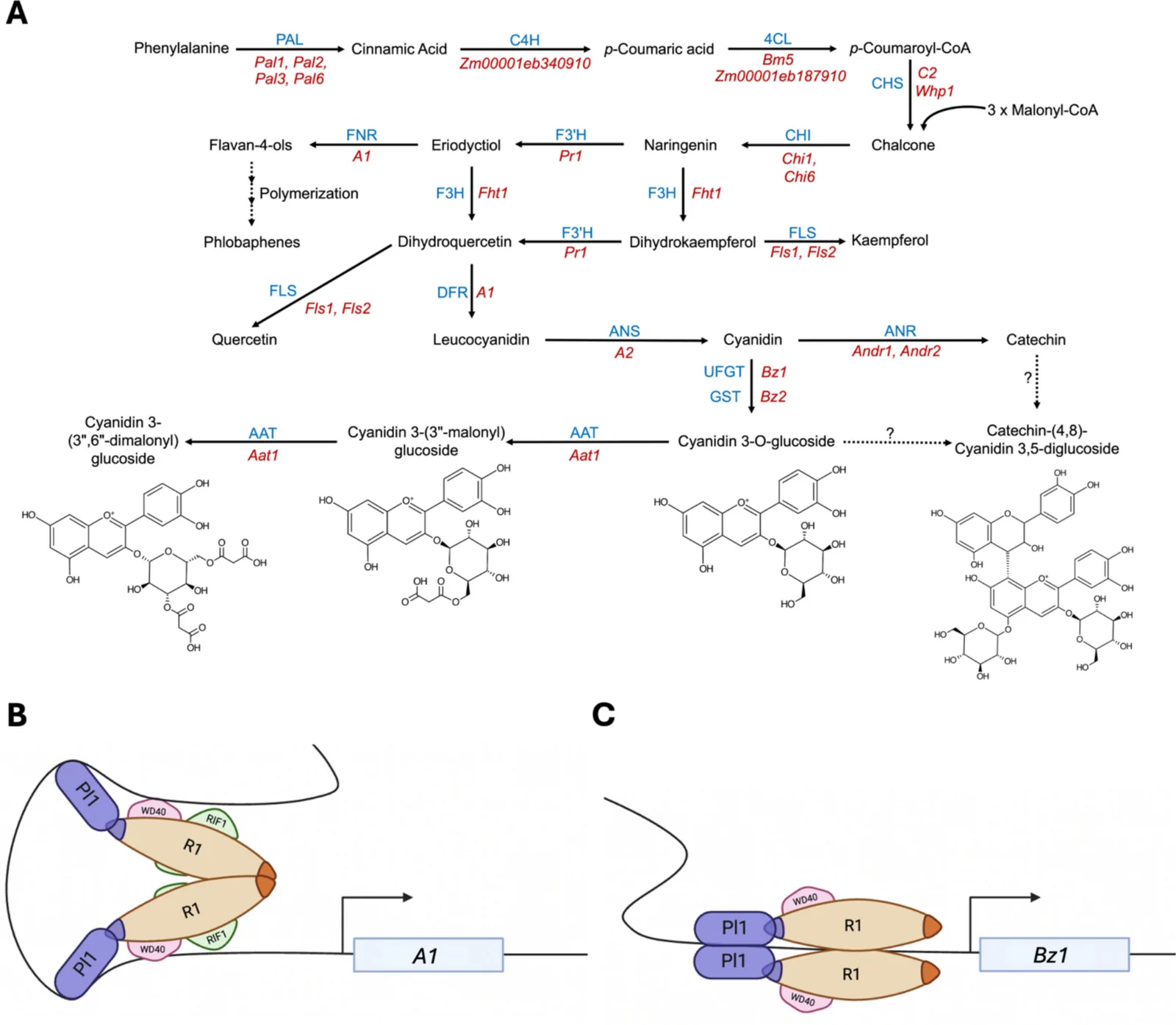
**(A)** A simplified depiction of the anthocyanin biosynthetic pathway in maize pericarp tissue. Structural genes are denoted in red and structural gene products are denoted in blue. PAL phenylalanine ammonia lyase; C4H cinnamate-4-hydroxylase; 4CL 4-Coumarate:CoA-ligase; CHS chalcone synthase; CHI chalcone isomerase; F3H flavonone 3-hydroxylase; F3’H flavonoid 3’-hydroxylase; DFR dihydroflavonol 4-reductase; LAR leucoanthocyanidin reductase; ANS anthocyanidin synthase; UFGT UDP-glucose flavonol glycosyltransferase; GST glutathione S-transferase; AAT anthocyanin acyltransferase. **(B**-**C)** Proposed anthocyanin biosynthetic regulatory factors in maize pericarp based on reports in maize aleurone. Illustrated assembly of **(B)** the core MBW complex (R1, PL1, and an unidentified WD40) and RIF1 (R-interacting factor 1) at the promoter region of the early anthocyanin biosynthetic gene, *A1*, and assembly of (**C**) the core MBW complex at the promoter region of the late anthocyanin biosynthetic gene, *Bz1*, once dimerization with RIF1 is disrupted. The anthocyanin biosynthetic pathway consists of structural genes (biosynthetic and transport genes) and can be divided into Early Biosynthetic Genes (EBGs) and Late Biosynthetic Genes (LBGs), demarcated by the synthesis of dihydroflavonol 4-reductase (DFR) (Fig. 1A) (Chatham et al. 2019; Khusnutdinov et al. 2021). Transcription of the maize DFR, *A1,* at the early stage requires assembly of the core MBW complex and *R-interacting factor 1* (*Rif1*), an EMSY-like protein involved in epigenetic, chromatin remodeling, at the *A1* cis-regulatory region through *Pl1* (Fig. 1B) (Hughes-Davies et al. 2003; Carey et al. 2004; Hernandez et al. 2007; Kong et al. 2012; Cappellini et al. 2021). It is predicted that homodimerization of *Lc1*’s ACT-like domains prevents dimerization at its bHLH site, allowing Rif1 to bind at the free bHLH domain. During LBG activation, disruption of dimerization at the ACT-like domain allows for bHLH homodimerization, reestablishing *Lc1*’s DNA-binding activity at the *Bz1* promoter (Fig. 1C) (Kong et al. 2012). Following anthocyanin biosynthesis, transport factors and ER-derived vesicles mediate cellular trafficking and storage of anthocyanins in the central vacuole (Manzoor et al. 2023). Two transport/storage genes, *Bz2* (*Bronze2*) and *Mrpa3* (*Multidrug resistance-associated protein3*), encoding a glutathione S-transferase (GST) and an ATP-binding cassette (ABC) transporter, respectively, have been characterized in the vacuolar localization of anthocyanins in maize aleurone (Marrs et al. 1995; Goodman et al. 2004)

A comprehensive survey of maize germplasm (Paulsmeyer et al. 2017) identified several donor landraces rich in anthocyanins and exhibiting pericarp pigmentation: Arequipa 35/Ames 8488 (North Central Regional Plant Introduction Station in Ames, IA, USA), “Inca Purple” (Amazonas Imports, Irwindale, CA, USA), and “Maize Morado” (Angelina’s Gourmet, Swanson, CT, USA). Elite maize inbreds with expired Plant Variety Protections (ex-PVP) and high heterotic expression were chosen to develop a breeding population (Hauck et al. 2014). Initial crosses were made in Summer 2014 between purple corn landraces and Midwestern ex-PVP inbreds: Arequipa 35 × B73, Arequipa 35 × Mo17, Inca Purple × LH123, and Maize Morado × PHG84. Near-isogenic color-converted lines were created in B73 and Mo17 backgrounds by backcrossing Arequipa 35 five times to the respective inbred, followed by at least five rounds of selfing. Kernel anthocyanin accumulation was selected for at each round of selection. The Amazonas inbred was generated from a cross (Inca Purple x LH123) then backcrossed once to LH123 followed by at least five rounds of selfing and selection for high anthocyanin accumulation in the pericarp tissue (Fig. 1). The Maize Morado inbred was generated from a cross (Maize Morado x PHG84) then backcrossed once to PHG84 followed by at least five rounds of selfing and selection for high anthocyanin accumulation in the pericarp tissue. Inbreds generated from these crosses will be referred to as B73 Color Converted, Mo17 Color Converted, Amazonas, and Maize Morado. None of the pigmented inbreds generated exhibited aleurone pigmentation (Fig. S1).

### Experiment 1 sample collection

**Fig. 2:**
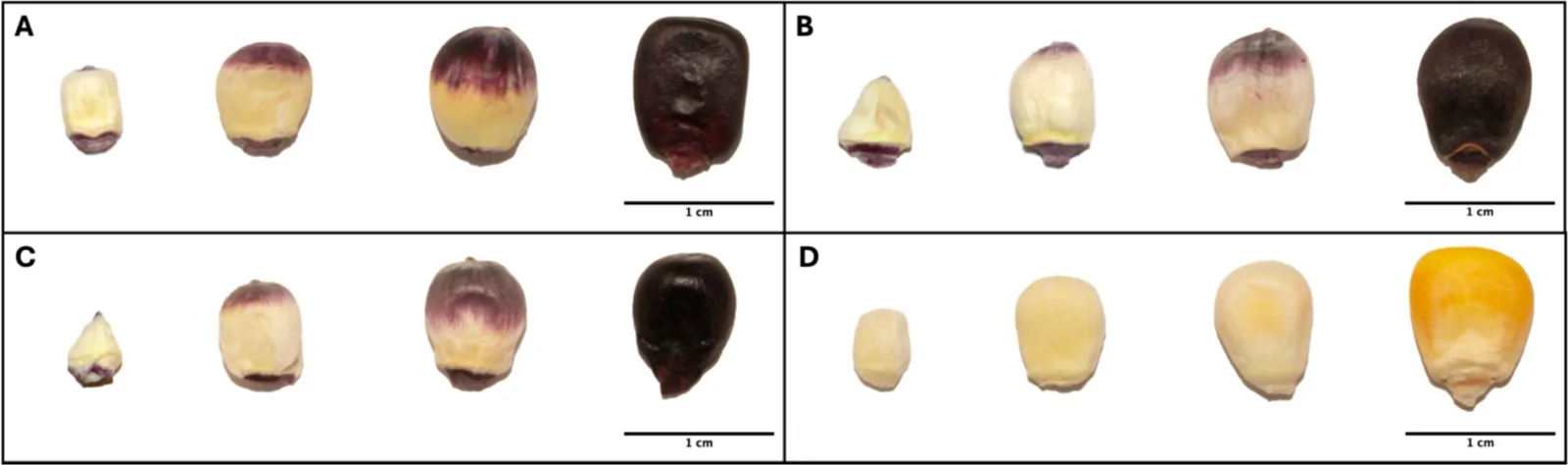
Freeze-dried kernels at 10, 15, and 20 DAP and mature dried kernels at 60 DAP, respectively. **(A)** B73 Color Converted. **(B)** Amazonas. **(C)** Maize Morado. **(D)** B73. Scale bar is set to 1 cm

Samples for Experiment 1 were collected at the University of Illinois Vegetable Crops Research farm in Champaign, IL, USA (40°04′36.1″N 88°14′24.2″W), in Summer 2021. Individual selfed ears of B73 Color Converted, Amazonas, and Maize Morado were harvested at 10, 15, and 20 DAP for a total of three biological replicates (ears) at each timepoint (Fig. 2). Three replicates of B73 were included as a negative control at each timepoint. All pigmented lines showed pigmentation initiation at the kernel tip, consistent with the findings that anthocyanin accumulation is initiated by the movement of the male gamete through the tip of the immature kernel via the pollen tube (Chatham et al. 2019). Pigmentation was found in the maternal pedicel of all pigmented inbreds and contributed to the majority of pigmentation in whole kernels seen at 10 DAP. Because our study focused on pigmentation in the pericarp tissue layer, forceps were used to effectively separate whole pericarp tissue from at least 20 kernels of each replicate for subsequent RNA-seq analysis. Extra care was taken to ensure that only pericarp tissue was collected at each timepoint for each genotype, so the amount of pericarp tissue exceeds aleurone contamination. Pedicel tissue was removed from each kernel to ensure RNA-seq was conducted exclusively on the pericarp tissue. Harvest times were chosen because it was possible to cleanly remove pericarp from the kernel without compromising the sample with aleurone or endosperm tissue. Pericarp tissue tends to separate from aleurone and endosperm tissues as the kernel develops. Pericarp tissue and additional intact, whole kernels were flash frozen and lyophilized for downstream analyses. Mature dry ears were harvested at 60 DAP (maturity) and dried down in a heated forced air dryer (35 °C) for at least 5 days.

### Experiment 2 sample collection

**Fig. 3:**
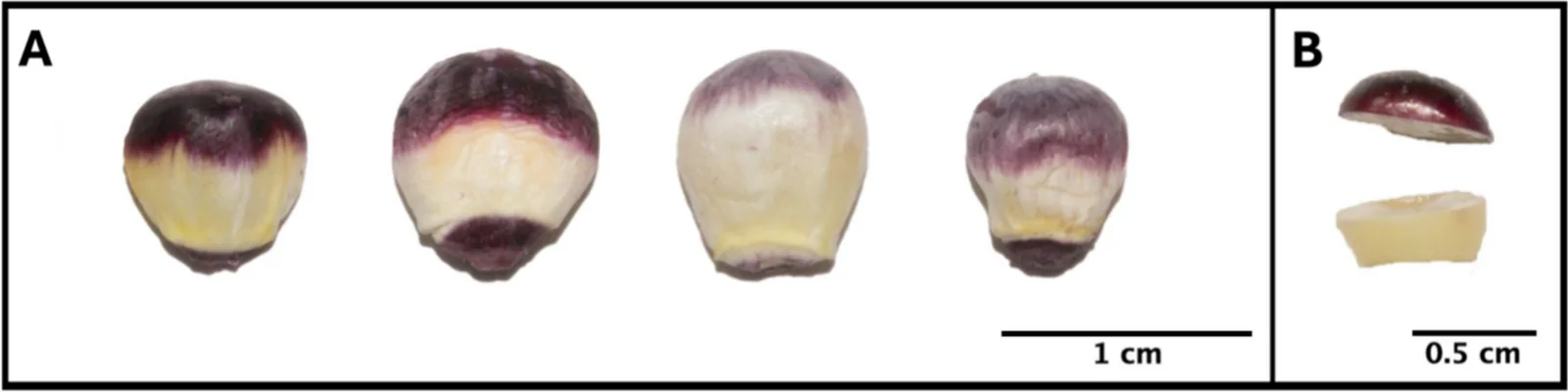
**(A)** Freeze-dried kernels at 18 DAP; (left to right) B73 Color Converted, Mo17 Color Converted, Amazonas, and Maize Morado. Scale bar is set to 1 cm. **(B)** Flash-frozen kernel excised into pigmented and unpigmented fractions prior to pericarp separation from aleurone and endosperm

Samples for Experiment 2 were grown in the same field location as Experiment 1 in the Summer of 2023. Kernels from B73 Color Converted, Mo17 Color Converted, Amazonas, and Maize Morado, were harvested at 18 DAP for a total of five biological replicates (ears). Ears were flash-frozen, and kernels were collected and excised at the boundary of pigmented and unpigmented pericarp using a razor blade (Fig. 3). Pigmented and unpigmented pericarp tissues were collected separately from at least 20 kernels of each replicate ear using forceps, following the same method used in Experiment 1. Unpigmented pericarp tissue fractions of the same kernels were harvested as a negative control. Following tissue separation, all samples were lyophilized and homogenized.

### Anthocyanin extraction

In Experiment 1, anthocyanins were extracted from three biological replicates (ears) of whole kernel tissue samples harvested in the Summer of 2021 at 10, 15, and 20 DAP and at kernel maturity (60 DAP). Mature kernels were homogenized using a coffee grinder prior to anthocyanin extraction. Lyophilized kernels were ground using metal beads. Using established anthocyanin extraction procedures (Chatham & Juvik 2021), lyophilized kernels were extracted using 25 mg of whole kernel tissue in a threefold dilution (375 µl) of 2% (*v/v*) formic acid. For mature kernels, 2 g of powder was extracted in 10 mL 2% (*v/v*) formic acid. In Experiment 2, freeze-dried pigmented kernel pericarp tissue fractions from five biological replicates (ears) of each purple corn inbred were used for quantification of anthocyanin content at 18 DAP. Due to the smaller available tissue volume, 1 mL of 2% (*v/v*) formic acid was added to 25 mg of tissue from each sample for a more effective extraction and to reduce variability between samples (Paulsmeyer and Juvik 2023b). Samples were incubated for 1 h at 50 °C. Samples were centrifuged and filtered following the protocol outlined in Experiment 1. All samples were incubated for 1 h at 50 °C. Samples were centrifuged (> 7500 *xg*) for 10 min, and the supernatant was filtered through a 0.45-µm PTFE filter.

### Ultra-high-performance liquid chromatography (UHPLC) analysis

Extracted anthocyanins were quantified on a Agilent 1290 Infinity II UHPLC (Agilent) using the settings outlined in (Paulsmeyer et al. 2022)). Samples were passed through a Poroshell 120 SB-C_18_ (3 mm × 100 mm, 1.9 μm) column (Agilent) at 50 °C. A linear dilution series of cyanidin 3-*O*-glucoside (C3G) standard (31.25, 62.5, 125, 250, 500, 1000 μg/ml) (MilliporeSigma) dissolved in 0.1% HCl in water was used to create a standard curve to estimate anthocyanin quantification. Major cyanidin-derived anthocyanins were defined in Paulsmeyer et al. (2017) (Fig. S2). In Experiment 1, anthocyanin contents were quantified as major anthocyanin-derived compounds per kernel (µg/dry kernel) using the average kernel weight of 10 kernels. In Experiment 2, anthocyanin contents were quantified as major anthocyanin-derived compounds per gram of dried pigmented pericarp tissue (µg/g DW).

### Statistical and data analysis

Anthocyanin concentration (µg/ml) per sample was quantified by converting anthocyanin-derived compound peak areas into C3G equivalents, using C3G standards and mass-spectrometry identified compounds (Paulsmeyer et al. 2022). In Experiment 1, anthocyanin content of whole kernels (µg/kernel) was calculated using Eq. 1.

1 $$ \begin{aligned} Anthocyanin content \left( {\mu g/\ker nel} \right) = & Anthocyanin concentration \left( {\frac{\mu g}{{ml}}} \right) \times \frac{{extraction solvent \left( {ml} \right)}}{sample weight \left( g \right)} \\ & \times \frac{{average \ker nel weight \left( {mg} \right)}}{\ker nel} \\ \end{aligned} $$

Mean separation was evaluated using a two-way ANOVA followed by the Tukey’s Honest Significant Differences (HSD) post hoc method. In Experiment 2, anthocyanin content of pigmented pericarp tissue fractions (µg/g DW) was calculated using Eq. 2.

2 $$ {\mathrm{Total}}\;{\mathrm{anthocyanin}}\;{\mathrm{content}} = \frac{{{\mathrm{Anthocyanin}}\;{\text{concentration }}\left( {\frac{{{\mathrm{mg}}}}{{{\mathrm{ml}}}}} \right){ } \times {\text{ extraction }}\;{\text{solvent }}\left( {{\mathrm{ml}}} \right)}}{{{\text{DW }}\left( {\mathrm{g}} \right)}} $$

Mean separation across genotypes was evaluated using a one-way ANOVA, followed by the Tukey’s Honest Significant Differences (HSD) post hoc method.

### RNA extraction and RNA-seq

RNA was extracted from at least 10 mg of homogenized pericarp tissue from three biological replicates of each line at each developmental timepoint. In Experiment 1, extractions were conducted on three biological replicates of each genotype at 10, 15, and 20 DAP for a total of 36 samples. In Experiment 2, extractions were conducted on three biological replicates of each genotype at 18 DAP for a total of 24 samples (12 pigmented samples and 12 unpigmented samples). Each sample underwent RNA extraction using a Quick-RNA™ MiniPrep kit (Zymo Research) following the manufacturer’s protocol with an on-column DNase I treatment step. All samples were submitted to the DNA Services laboratory of the Roy J. Carver Biotechnology Center at the University of Illinois at Urbana-Champaign for evaluation and processing. Libraries were constructed with a Kapa HyperPrep mRNA kit (Roche) and PCR-amplified with the Kapa HiFi polymerase (Roche). In Experiment 1, all 36 libraries were sequenced on an Illumina NovaSeq 6000 S4 flowcell (Illumina) using paired-end 150 bp fragments. In Experiment 2, all 24 libraries were pooled and sequencing of paired-end 150 bp fragments was conducted on an Illumina NovaSeq X Plus 10B flowcell (Illumina). FASTQ files were demultiplexed using the bcl2fastq v2.20 Conversion Software (Illumina).

### RNA-seq analysis

Adaptor sequences were trimmed, and quality was assessed via FastQC by the DNA Services laboratory. Reads with an average quality score > 28 were aligned to the Zm-B73-REFERENCE-NAM-5.0 reference genome using two-pass Spliced Transcripts Alignment to a Reference (STAR) 2.7.10a alignment (Dobin and Gingeras 2015; Woodhouse et al. 2021). A summary of read counts for Experiment 1 and 2 is listed in Table S1 and S2, respectively. Reads were quantified and normalized in Transcripts Per Million (TPM) using RNA-Seq by Expectation Maximization (RSEM) (Li & Dewey 2011). Genes that did not meet the filtering threshold (TPM ≤ 0.1 in at least one sample) were considered noise and removed prior to differential gene expression (DGE) analysis with limma-trend (Law et al. 2014). Limma-trend was implemented to fit a linear model to each gene and to calculate DGE using a *t* test for specified contrasts. The DGE analysis compared whole pericarp tissue of each pigmented line to whole pericarp tissue of an unpigmented negative control (B73). Candidate genes were identified as having significant DGE (FDR *p* ≤ 0.01 and |log_2_-fold-change|≥ 1).

### Determining fixed loci in the B73 color converted line using bulk segregant analysis

Variant calling was performed in BCFtools v1.2 (Bonfield et al. 2021; Danecek et al. 2021) using the multiallelic calling model (Danecek et al. 2016). All biological replicates of B73 Color Converted and B73 from each time point were bulked with their respective genotype for a combined total of 9 replicates per genotype. The genomic VCF file was passed through the “VariantsToTable” plugin in GATK to be filtered in the *R* package “QTLseqr” (Mansfeld and Grumet 2018). Sites with non-reference calls in B73 were removed as they may be errors in alignment. Sites were filtered to a minTotalDepth of 50 and a minSampleDepth of 10. This resulted in 32,390 high-quality SNPs. Variants were detected across the entire genome, which is most likely artifact of sequence misalignment. A sliding window of 1 Mb was applied to count the number of SNPs in a region and is reported in Figure S4. The Bayesian Bulk Segregant Analysis was performed using the method by Liu et al. 2012. Dense SNP regions were observed near known anthocyanin biosynthetic genes. On the basis of this, we assume that fixed alleles are present in these regions of the pigmented NIL compared to its recurrent parent.

### Weighted gene correlation network analysis (WGCNA)

Unlike Experiment 1 where a separate negative control was harvested (B73), Experiment 2 utilized the unpigmented pericarp tissue fraction of the same kernel for DGE analysis. The same DGE threshold was set as in Experiment 1 (FDR *p* ≤ 0.01 and |log_2_-fold-change|≥ 1). Pericarp-pigmented fractions underwent a Weighted Gene Correlation Network Analysis (WGCNA) in R using the WGCNA package (Langfelder and Horvath 2008) to cluster expressed genes based on similar expression trends, under the assumption that similarly expressed genes may belong to related biochemical pathways. Anthocyanins were absent in unpigmented tissue samples; thus, they were excluded from WGCNA analysis. Hierarchical clustering organized DEGs into 10 gene modules based on similar gene expression patterns. Each module is characterized by a module eigengene value (MEV) representative of the general expression trend of all genes within the module. A sample’s MEV denotes its overall expression of all genes within the module. A MEV was calculated from all 10 gene modules for each sample, resulting in 120 MEVs. Pearson correlation analysis of MEVs against the anthocyanin content (µg/g DW) of each sample identified significantly correlated clusters. A subsequent Spearman correlation analysis was conducted on modules that did not meet the assumptions of normality. Gene module membership and gene trait significance, representing the strength of association between a gene and the entire module and the strength of association between a gene and anthocyanin content, respectively, were calculated according to the WGCNA software tutorials (Langfelder and Horvath 2008). Candidate genes for anthocyanin biosynthesis from significant modules were defined by a DEG in at least one genotypic comparison and high gene module membership and gene trait significance. Gene module membership and gene trait significance, representing the strength of the correlation between a gene and a module and the strength of the correlation between a gene and anthocyanin content, respectively, were calculated. Kyoto Encyclopedia of Genes and Genomes (KEGG) pathway enrichment analysis was computed through ShinyGO v.0.80 (Ge et al. 2020; Kanehisa et al. 2023) using a hypergeometric test (FDR *p* ≤ 0.05) to characterize module identity.

## Results

### Experiment 1: Transcriptomics of whole kernel pericarp tissue across kernel development

#### Ultra-high-performance liquid chromatography (UHPLC) quantified differences in anthocyanin content across kernel development

**Fig. 4:**
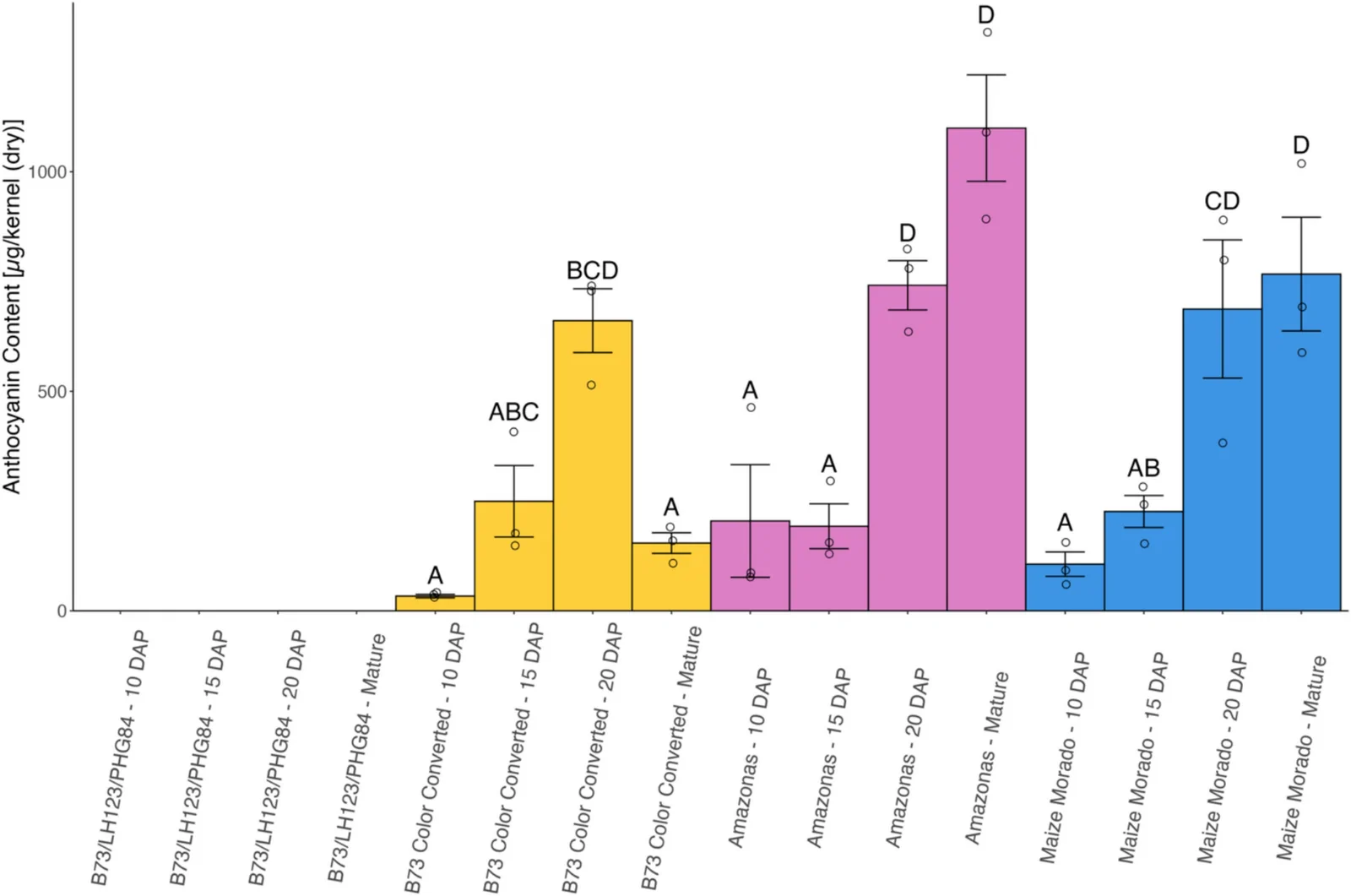
Anthocyanin content [µg total anthocyanins/kernel (dry)] of whole kernel tissue of each genotype (left to right, B73/LH123/PHG84, B73 Color Converted, Amazonas, and Maize Morado) at each timepoint (left to right, 10, 15, and 20 DAP and maturity). All elite inbreds (B73, LH123, and PHG84) are grouped together due to the absence of anthocyanin content. Three biological replicates of each genotype are displayed. Error bars indicate standard error. Different letters denote significantly different means according to Tukey’s HSD (*p* value ≤ 0.05)

Significant differences in the concentrations of anthocyanins were seen across different genotypes and harvest dates (Fig. 4). Anthocyanin content ranges from 25 ± 3.94 µg/kernel (dry) (B73 Color Converted at 10 DAP) to 1315 ± 121 µg/ kernel (dry) (Amazonas Mature Dry). All ex-PVP inbreds (B73, LH123, and PHG84) contained no detectable anthocyanins. Amazonas and Maize Morado exhibited a significant spike in anthocyanin content at from 15 to 20 DAP, while B73 Color Converted exhibited an increase that was not statistically significant.

#### Differential expression of structural genes across kernel development

**Fig. 5:**
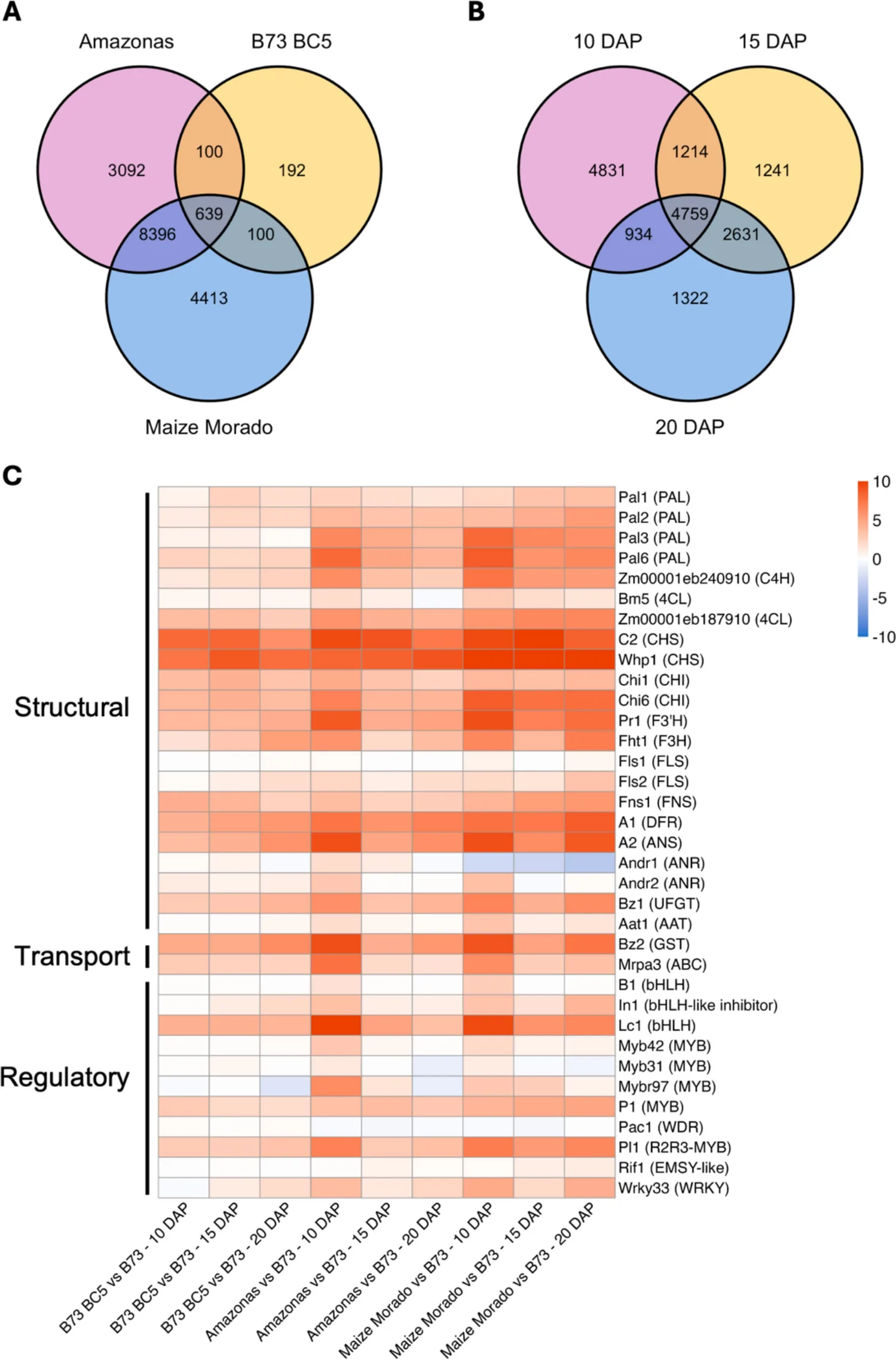
Venn diagram of **(A)** total DEGs identified from each pigmented genotype compared to B73 and **(B)** total DEGs of all pigmented genotypes at each developmental timepoint compared to B73. Overlapping regions indicate shared DEGs. **(C)** Log_2_ fold-change expression of putative anthocyanin biosynthetic structural, transport, and regulatory genes. Expression was measured as the difference in gene expression [Transcripts per Million (TPM)] between whole pericarp tissue of each pigmented genotype and B73 at 10, 15, and 20 DAP. Blue indicates the level of downregulation and red indicates the level of upregulation. Gene products are listed in parentheses

RNA-seq analysis was conducted on whole pericarp tissue from three pericarp-pigmented genotypes: B73 Color Converted, Amazonas, and Maize Morado, at 10, 15, and 20 DAP. Across the development of all pigmented genotypes, 7,650 DEGs were identified when compared to B73 (FDR *p* ≤ 0.01): 324 (B73 Color Converted), 4371 (Amazonas), and 5754 (Maize Morado) (Fig. 5A). Putative anthocyanin biosynthetic genes, based on previous reports, were categorized as structural, regulatory, or transport/storage genes, and expression levels were measured (Fig. 5C) (Chatham et al. 2019; Chatham and Juvik 2021; Woodhouse et al. 2021; Paulsmeyer and Juvik 2023b). A DGE analysis was performed on all purple varieties compared to unpigmented B73 and is included in Table S3. In general, EBGs and LBGs were upregulated across all of the pigmented kernel development stages. Specifically, 4-Coumarate:CoA ligase (4CL) Zm00001eb187910, chalcone synthase (CHS) genes *Colorless2* (*C2*) and *White pollen1* (*Whp1*), two chalcone isomerase (CHI) genes *Chalcone isomerase1* (*Chi1*) and *Chalcone flavanone isomerase6* **(***Chi6*), flavonoid 3'-hydroxylase (F3'H) gene *Red aleurone1* (*Pr1*), DFR *A1,* anthocyanidin synthase (ANS) gene *Anthocyaninless2* (*A2*), and UDP glucose: flavonoid 3-*O*-glucosyltransferase *Bz1*, were differentially expressed across all developmental timepoints for pigmented genotypes (Fig. 1A, Table S3). Anthocyanin activation occurred earlier in Amazonas and Maize Morado, with the EBGs being upregulated higher at 10 DAP relative to the remaining timepoints (Fig. 5C, Table S3). Of the known anthocyanin biosynthetic transport genes, only *Bz2* was differentially expressed across the development of all pigmented genotypes. *Mrpa3* was upregulated in Maize Morado and in B73 Color Converted at all harvest dates and Amazonas at 10 DAP.

#### Differential expression of regulatory genes across kernel development

Of the predicted anthocyanin biosynthetic regulatory genes, the MBW complex genes, *Pl1* and *Lc1,* yielded significant DGE across the development of all pigmented genotypes. *B1*, another candidate bHLH functioning in the aleurone MBW complex, was not highly expressed (≤ 6.13 TPM) in any sample. Expression of *Colored aleurone1* (*C1*), the Myb member functioning in the aleurone MBW complex, was not detected (TPM ≤ 0.1) in any sample. *Pac1*, encoding a WD40 protein known to activate aleurone pigmentation, was not significantly differentially expressed, but was expressed in all samples (48.79–128.84 TPM) (Table S3). The gene model for *P1* was not differentially expressed and had low expression (≤ 0.33 TPM). Based on the upregulation of maysin biosynthetic genes, this was unexpected. Upon further investigation, it appears there are 11 tandem duplications of *P1* in the B73 genome (Goettel and Messing 2009) and eight gene models with > 99% similarity (Fig. S3). STAR aligned most *P1*-like transcripts to *Mybr102*, which is 86 kb away and 100% identical to *P1*. The *P1* gene model appears to be annotated incorrectly as it is truncated by 469 bp in relation to *Mybr102*. Since the origin of the cDNA sequence cannot be determined, the summation of the eight duplicate gene models was used to represent *P1*. Given this, *P1* was expressed 1.86- to 4.95-fold higher than B73 in every sample (Table S3). In addition to these activator genes, several repressor genes were expressed in pericarp. *Mybr97* is a gene that is known to decrease anthocyanin content in vegetative tissues (Paulsmeyer and Juvik 2023b). This gene was expressed in all samples, with the most extreme expression at 10 DAP in Amazonas (Fig. 5C). Furthermore, *Intensifier1* (*In1*), *Myb8*, *Myb31,* and *Myb42* are known repressors of early biosynthetic genes (Fornalé et al. 2006). *Myb8* was upregulated in Amazonas and Maize Morado, but not B73 Color Converted. The remaining repressors were not significantly differentially expressed overall but were expressed in every sample (Fig. 5C).

#### Differential expression of candidate anthocyanin biosynthetic genes across kernel development

Overexpression analysis of the top 10 DEGs across the development of pigmented genotypes revealed consistent upregulation of anthocyanin biosynthetic genes, *C2* and *Whp1*, as well as additional candidate genes, *Jacalin6* (*Jac6*) and *UDP-glucosyl transferase1* (*Ugt1*) (Table 1). While not previously characterized in the anthocyanin biosynthetic pathway, consistent upregulation of *Jac6* and *Ugt1* suggests their influence over anthocyanin production. The identification of the multidrug and toxic compound extrusion (MATE) transporter, *Mate12*, in B73 Color Converted suggests an alternative mechanism for anthocyanin transport. Shared overexpression of *beta-glucosidase9* (*glu9*), a candidate anthocyanase, in B73 Color Converted and Maize Morado may act as a anthocyanin degradation factor, influencing anthocyanin accumulation (Barbagallo et al. 2007). Significant differential expression of *glu9* was absent in Amazonas (Table S4).

**Table 1:** Ten highest DEGs according to log_2_-fold change of each pigmented genotype compared to B73. Gene model corresponds to the Zm-B73-REFERENCE-NAM-5.0 reference genome

Gene model	Gene locus symbol	Protein product associated with gene locus	Log_2_-fold change	False discovery rate
*B73 Color Converted*				
Zm00001eb392870	*Jac6*—*jacalin6*	Jacalin	8.12	4.45E − 22
Zm00001eb113440	*Whp1*—*white pollen1*	Chalcone synthase	8.06	8.76E − 20
Zm00001eb077490	*Glu9*—*beta-glucosidase9*	Beta glucosidase	7.82	6.09E − 18
Zm00001eb280930	*Ugt1*—*UDP-glucosyl transferase1*	UDP-glucuronosyl/UDP-glucosyltransferase	7.75	1.54E − 18
Zm00001eb198030	*C2*—*colorless2*	Chalcone synthase	7.45	4.95E − 20
Zm00001eb197860	*Mads52*—*MADS-transcription factor 52*		5.95	2.36E − 26
Zm00001eb048870	*Mate12*—*multidrug and toxic compound extrusion12*	MATE	5.76	1.76E − 15
Zm00001eb048110	*Bz2*—*bronze2*	Glutathione S-transferase, BZ2	5.28	1.40E − 07
Zm00001eb159020	*A1*—*anthocyaninless1*	NADPH dihydroflavonol reductase	5.03	1.18E − 13
Zm00001eb105580	*Sm2*—*salmon silk2*	Rhamnosyl transferase	5.01	2.44E − 10
*Amazonas*				
Zm00001eb113440	*Whp1*—*white pollen1*	Chalcone synthase	8.66	7.50E − 22
Zm00001eb149470			8.82	5.85E − 22
Zm00001eb197860	*Mads52*—*MADS-transcription factor 52*		8.09	1.60E − 31
Zm00001eb198030	*C2*—*colorless2*	Chalcone synthase	8.62	4.75E − 23
Zm00001eb276520			8.05	1.90E − 32
Zm00001eb280930	*Ugt1*—*UDP-glucosyl transferase1*	UDP-glucuronosyl/UDP-glucosyltransferase	8.44	9.08E − 21
Zm00001eb353970			9.36	3.99E − 23
Zm00001eb392870	*Jac6*—*jacalin6*	Jacalin	8.63	5.59E − 24
Zm00001eb408780			8.22	2.59E − 32
Zm00001eb421480			8.24	4.82E − 29
*Maize Morado*				
Zm00001eb113440	*Whp1*—*white pollen1*	Chalcone synthase	11.20	2.77E − 25
Zm00001eb077490	*Glu9*—*beta-glucosidase9*	Beta glucosidase	10.36	8.72E − 23
Zm00001eb392870	*Jac6*—*jacalin6*	Jacalin	10.32	2.21E − 26
Zm00001eb280930	*Ugt1*—*UDP-glucosyl transferase1*	UDP-glucuronosyl/UDP-glucosyltransferase	10.17	2.78E − 23
Zm00001eb421480			9.36	5.88E − 31
Zm00001eb198030	*C2*—*colorless2*	Chalcone synthase	9.31	3.63E − 24
Zm00001eb105580	*Sm2*—*salmon silk2*	Rhamnosyl transferase	8.89	1.56E − 18
Zm00001eb376040	*Ufgt3*—*UDP-flavone-glycosyltransferase3*	Flavone 5-O-glucosyltransferase	8.30	7.20E − 20
Zm00001eb276520			8.26	3.60E − 33
Zm00001eb229190	*A2*—*anthocyaninless2*	Leucoanthocyanidin dioxygenase	8.20	4.98E − 13

#### Flux through various flavonoid pathways

The various outputs of the flavonoid biosynthetic pathway, such as anthocyanins, phlobaphenes, flavones, and flavonols, are known to share precursors (Fig. 1A). While overexpression of anthocyanin biosynthetic genes was seen across pigmented genotypes, flux through various flavonoid biosynthetic pathways was seen simultaneously. Phlobaphenes are water-insoluble, brick red pigments that are the result of polymerized flavan-4-ols. The biosynthesis of these molecules is activated by *Pericarp color1* (*P1*), a pericarp-specific Myb-like gene. Flavones are another off-shoot of flavan-4-ols that are activated by *P1*. Maysin is the most notorious C-glycosyl flavone since has long been known to confer plant resistance to certain herbivores (Casas et al. 2016). Biosynthesis of maysin is catalyzed by four enzymes: *Flavanone 2-hydroxylase* (*Fns1*), *C-glucosyl transferase1* (*Cgt1*), *Salmon silks1* (*Sm1*), and *Sm2* (Morohashi et al. 2012). Notably, a candidate maysin-related gene was significantly overexpressed in Maize Morado, *UDP-flavone-glycosyltransferase3* (*Ufgt3*), suggesting additional enzymes may enhance maysin biosynthesis. A functional *P1* must be active in the pigmented varieties since the entire maysin pathway was upregulated in every sample. Flavonols, such as quercetin and kaempferol, form an additional off-shoot of the flavonoid biosynthetic pathway. This requires either *Flavonol synthase1* (*Fls1*) or *Flavonol synthase2* (*Fls2*) (Fig. 1A). *Fls2* was not differentially regulated in pericarp, but *Fls1* was upregulated in Maize Morado and in Amazonas at 10 and 15 DAP.

Anthocyanins are able to interact with various flavonoids, generating increased chemical diversity. A specific class of anthocyanins which are of note are the condensed forms. These are anthocyanins conjugated with flavan-3-ols and are present in all varieties chosen for Experiment 1, with Maize Morado containing the largest quantity (Fig. S2). The biosynthetic pathway of these compounds is unknown. Interestingly, all condensed forms contain 3,5-diglucoside anthocyanins, when the predominant form of anthocyanins produced is 3-glucosides (Chatham et al. 2018). *Ugt1* was consistently upregulated in every purple variety and could be a gene candidate for further investigation (Table 1). Flavan-3-ols are synthesized by either the *Anthocyanidin reductase1* (*Andr1*) or *Anthocyanidin reductase2* (*Andr2*) enzymes. However, *Andr1* was only upregulated in Amazonas 10 and 15 DAP and downregulated in Maize Morado. *Andr2* was significantly upregulated in Amazonas and Maize Morado at 10 DAP.

#### Identifying fixed loci responsible for anthocyanin accumulation in the B73 color converted line

**Fig. 6:**
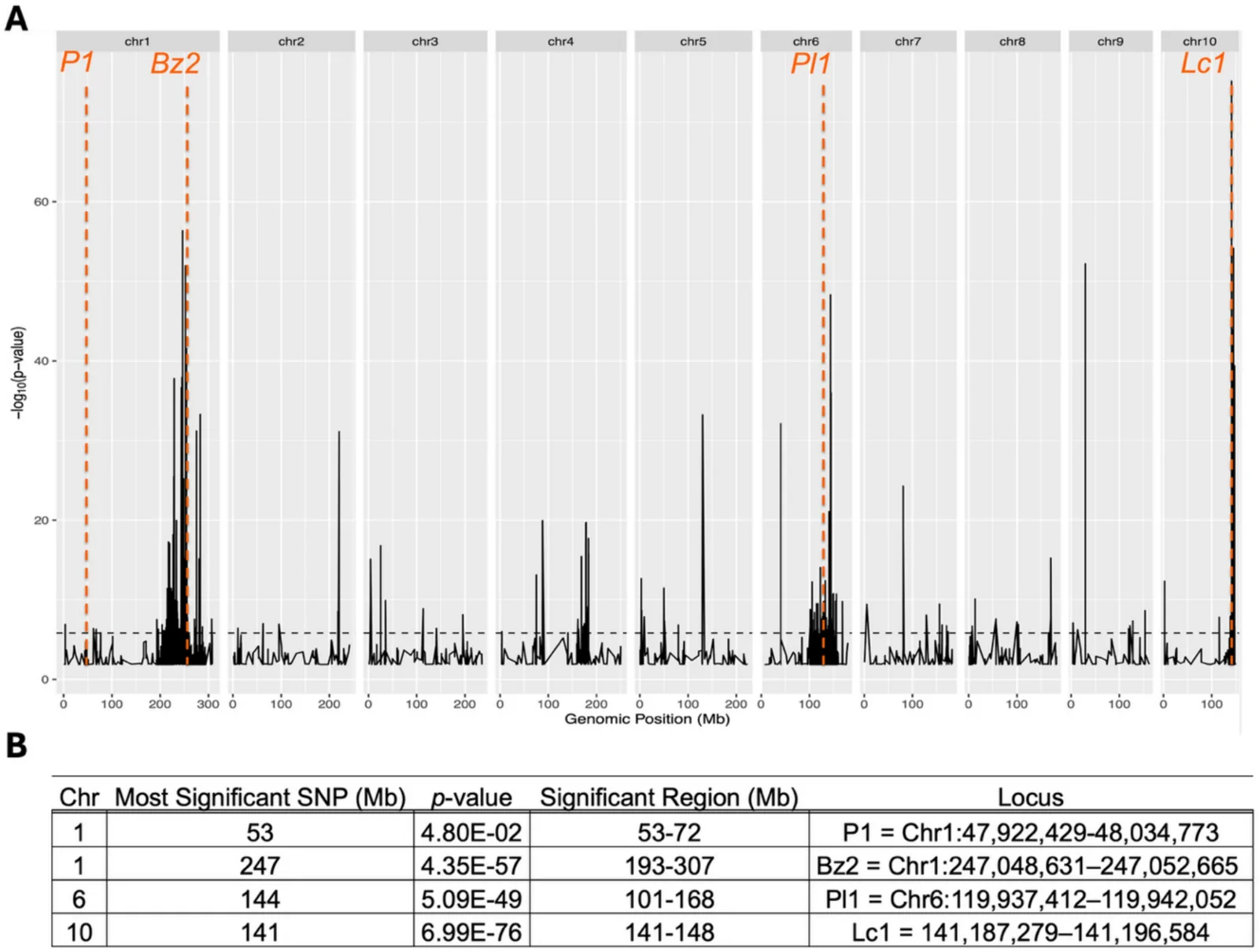
Bulk Segregant Bayesian Analysis of B73 Color Converted compared to B73. **(A)** The y-axis represents the probability that SNP genomic sequences in B73 Color Converted are significantly different from B73 [ − log_10_(*p* value)]. The horizontal dashed line represents the Bonferroni adjusted *p* value. Several regions of significant SNPs in B73 Color Converted are aligned with known anthocyanin-related genes from the Zm-B73-REFERENCE-NAM-5.0 reference genome and are indicated with vertical lines. **(B)** Chromosomal and genomic locations of the most significantly different SNPs and their significance levels in B73 Color Converted. The size of the significant region of SNPs is determined by the Bonferroni adjusted *p* value threshold. Known anthocyanin-related loci that fall within these significant regions are listed

The B73 Color Converted line underwent five rounds of backcrossing before selfing. In the absence of selection for increased anthocyanin content, theoretically, the inbred isoline is 96.875% (1 – ( ½)^5^) similar to reference inbred B73. Genetic variants were called using the aligned transcripts compared to B73. The number of SNPs in a 1 Mb window across the genome is reported in Figure S4. SNPs appear to be distributed throughout the genome, which may be an artifact of sequence misalignment. For a more rigorous analysis of SNP fixation, a Bayesian Bulk Segregant Analysis was performed on filtered variants using the method by Liu et al. 2012. The full results of the analysis are presented in Figure S4. Major transcriptional regulators *Lc1* and *Pl1* were significantly fixed in B73 Color Converted according to the Bayesian analysis with a Bonferroni threshold, as determined by the large density of SNPs located near these loci across all nine B73 Color Converted samples (Fig. 6). *P1* did not contain any significant SNPs, but appears to be fixed based on the relatively large number of variants near the gene (Figure S4). In addition to these loci, a major section of the distal end of Chromosome 1 was significantly fixed in B73 Color Converted. Within this region is *Bz2*, but no other anthocyanin-related genes are differentially regulated. Many developmental and domestication genes are within the locus, which might explain why the region is fixed. These genes include *Brachytic2* (*Br2*), *Brassinosteroid-deficient dwarf1*, *Defective kernel35*, *Dwarf plant8*, *Empty pericarp4*, *Empty pericarp10*, *Empty pericarp18*, *Empty pericarp21*, *Knotted1*, *Root hair defective1*, *Teosinte branched1*, *Unbranched2* (*Ub2*), *Vestigial glume1*, *Viviparous8*, and *Viviparous14*. *Br2* and *Ub2* were significantly upregulated in B73 Color Converted. Many other SNPs were significant in the Bayesian analysis. Chromosome 4 contained two loci with significant SNPs spanning more than 1 Mb (Fig. 6). Many other SNPs were significant but were within relatively smaller windows (< 1 Mb). These may be fixed in B73 Color Converted, but no major anthocyanin genes were found underlying the SNPs.

### Experiment 2: transcriptomic analysis of pigmented and unpigmented pericarp kernel tissue fractions at 18 DAP

A substantial increase in anthocyanin content was observed across all genotypes between 15 and 20 DAP (Fig. 4). To identify candidate genes underlying the spike in anthocyanin content, the transcriptome of pigmented and unpigmented pericarp were harvested at 18 DAP and compared; pigmented and unpigmented pericarp fractions were harvested from the same kernels, providing a robust negative control. An additional pericarp-pigmented line, Mo17 Color Converted, was included as a second near-isogenic line sharing the same pigmented, recurrent parent (Arequipa 35) with B73 Color Converted.

#### UHPLC quantified anthocyanin content of pigmented pericarp tissue fractions at 18 DAP

**Fig. 7:**
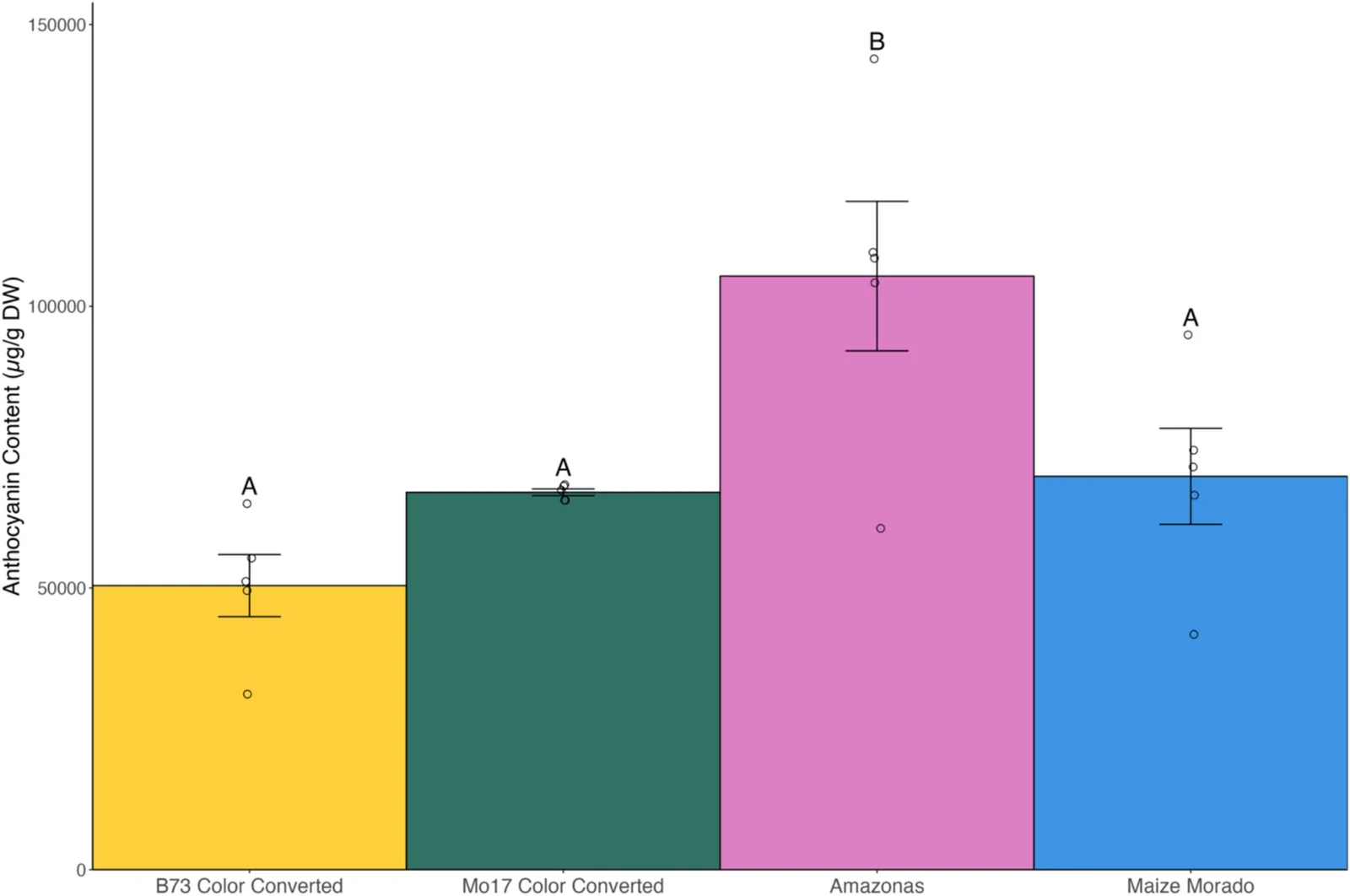
Anthocyanin content (µg total anthocyanins/g dry weight (DW)) of pigmented pericarp tissue fractions of each genotype (left to right, B73 Color Converted, Mo17 Color Converted, Amazonas, and Maize Morado) at 18 DAP. Five biological replicates of each genotype are displayed. Error bars indicate standard error. Different letters denote significantly different means according to Tukey’s HSD (*p* value ≤ 0.05)

Anthocyanin content was measured from the pigmented pericarp tissue of four pericarp-pigmented inbreds: B73 Color Converted, Mo17 Color Converted, Amazonas, and Maize Morado. Average anthocyanin content of pigmented pericarp tissue fractions ranged from 50,000 ± 5500 µg/g DW (B73 Color Converted) to 110,000 ± 13,000 µg/g DW (Amazonas) (Fig. 7). Anthocyanin levels were not detected in unpigmented pericarp tissue fractions. Amazonas exhibited a higher anthocyanin content relative to the other pigmented genotypes, likely attributable to its thicker pericarp (Fig. S1). Maize Morado exhibited enrichment of condensed forms, demonstrating additional compositional diversity of these two genotypes.

#### Differential expression of putative anthocyanin biosynthetic, transport, and regulatory genes in pigmented pericarp relative to unpigmented pericarp of the same kernels at 18 DAP

As expected, the core regulatory genes *Lc1* and *Pl1* were upregulated in all samples. *P1* was expressed, but not significantly differentially expressed. A gene candidate for anthocyanin LBG activation, *Wrky33,* was also upregulated in every sample. Interestingly, many known repressor genes were also upregulated. *In1* and *Mybr97* were upregulated in every sample with expression up to 6.62-fold higher for *In1* and up to 4.80-fold higher for *Mybr97*. *Myb42* was exclusively upregulated in Amazonas and Maize Morado. The full list of upregulated genes is included in Table S5.

**Fig. 8:**
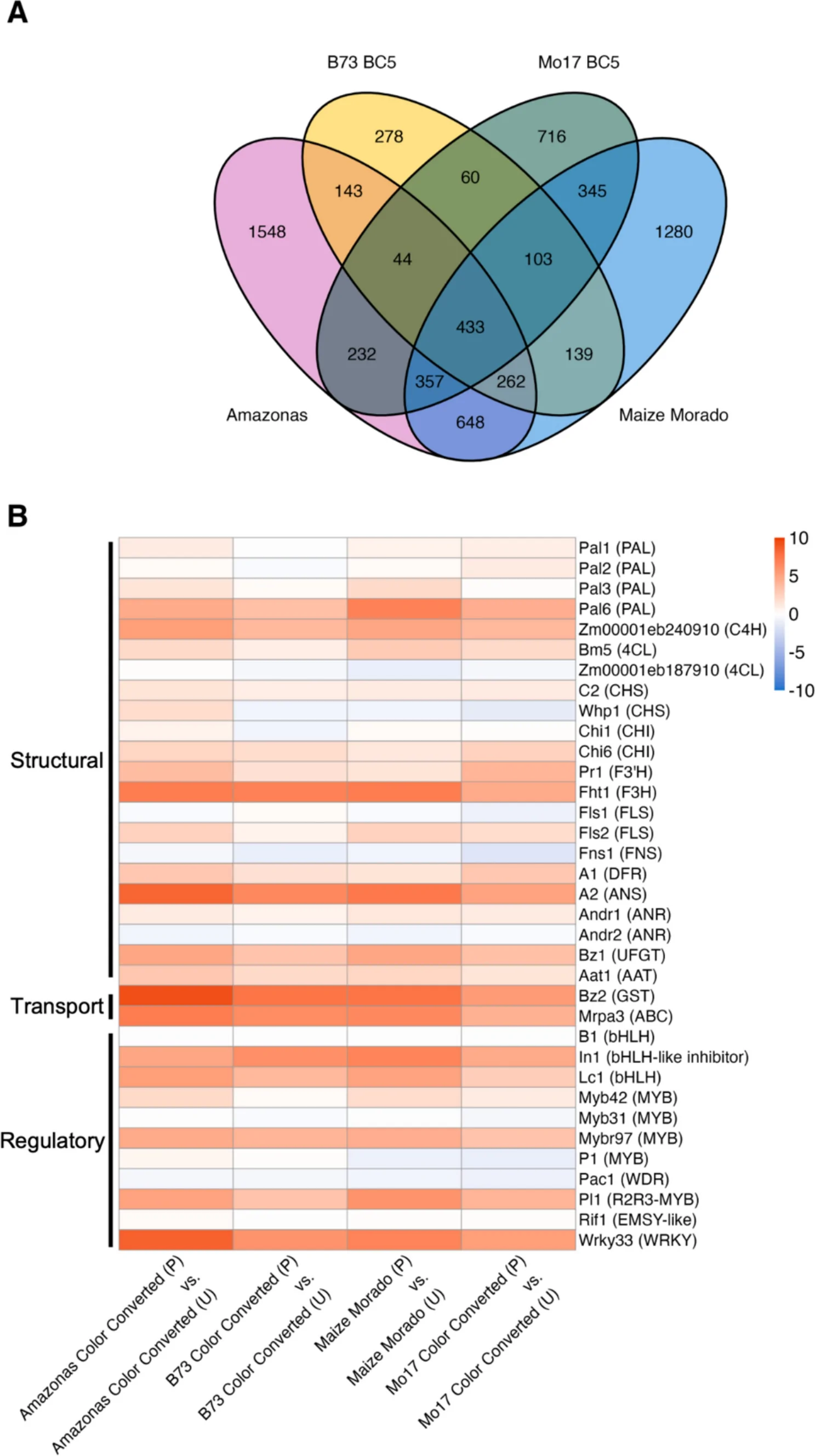
**(A)** Venn diagram of total DEGs identified from each genotype at 18 DAP. DEGs were identified from their upregulation (FDR *p* ≤ 0.01) in pigmented pericarp relative to unpigmented pericarp. **(B)** Log_2_ fold-change expression of putative anthocyanin biosynthetic structural, transport, and regulatory genes. Expression was measured as the difference in gene expression [Transcripts per Million (TPM)] between pigmented (P) and unpigmented pericarp (U) of the same kernels at 18 DAP. Red depicts upregulation and blue depicts downregulation. Gene products are listed in parentheses

Comparisons of pigmented pericarp to unpigmented pericarp of the same inbred genotype identified DEGs (FDR *p* ≤ 0.01): 1462 (B73 Color Converted), 2290 (Mo17 Color Converted), 3566 (Maize Morado), and 3667 (Amazonas) (Fig. 8A). Once again, the near-isogenic lines (NILs) showed fewer unique DEGs associated with pericarp when compared to the RILs. Among these DEGs, a subset corresponded to putative anthocyanin biosynthetic genes (Fig. 8B, Table S6). Of the predicted structural genes, *Pal6*, *C4H* (Zm00001eb240910), *Flavanone 3-hydroxylase1* (*Fht1*), *Pr1, A2, Bz1,* and *Anthocyanin acyltransferase1* (*Aat1*) were significantly upregulated across all pigmented tissue fractions (Fig. 8B). The known transporters, *Bz2* and *Mrpa3*, were also significantly upregulated in the pigmented pericarp fractions of all genotypes (Fig. 8B). Select genes displayed consistent expression in both pigmented and unpigmented tissues and were not differentially expressed. These included anthocyanin biosynthetic genes, *C2*, *Chi1*, *Pal1*, *Pal2*, and *Whp1*.

#### Top DEGs of each genotype were strongly correlated with anthocyanin content

**Fig. 9:**
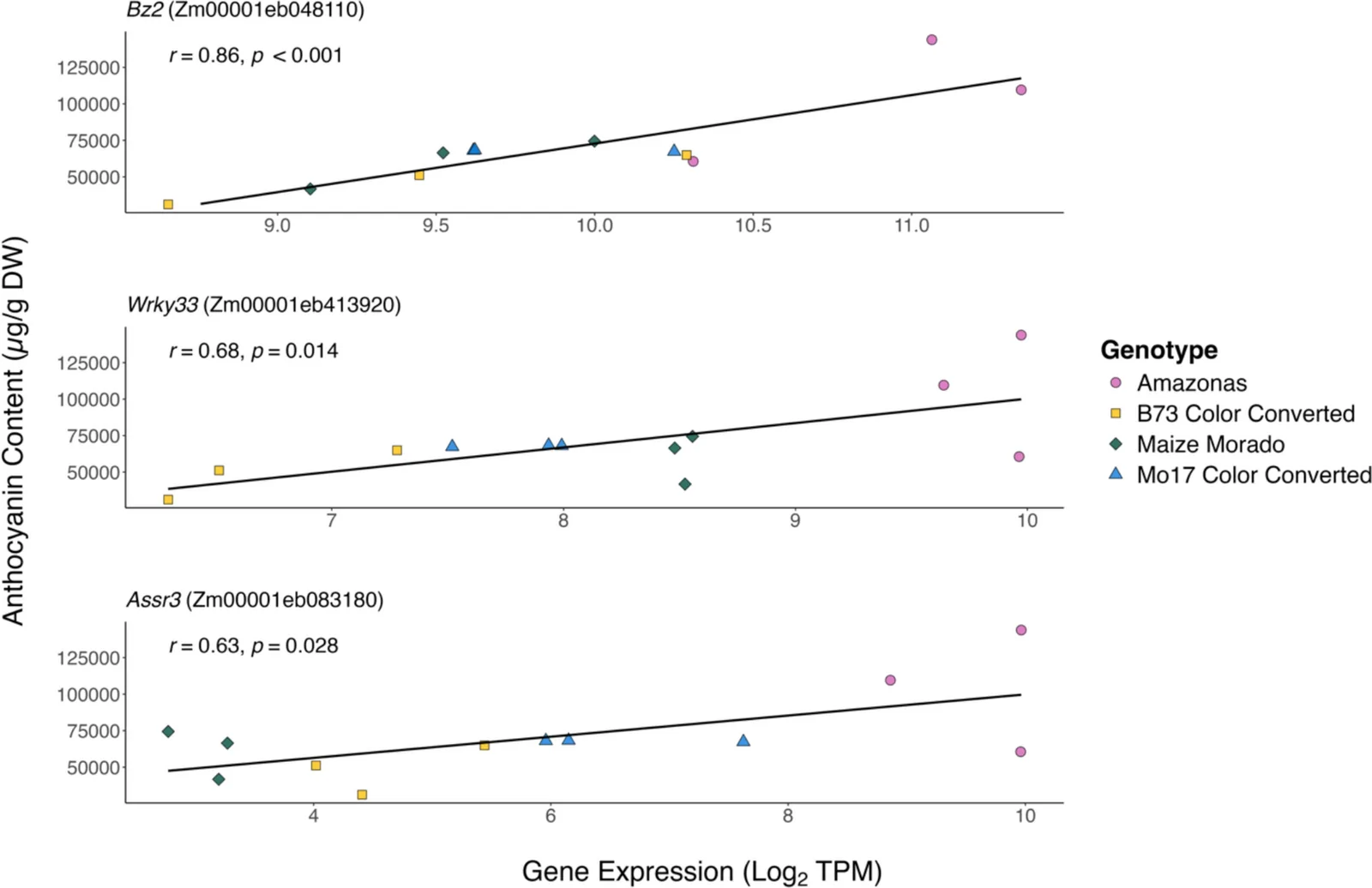
Significant gene correlations (*p* value ≤ 0.05) from Table 2 with the greatest expression in Amazonas, exhibiting the highest concentration of pericarp anthocyanins. Pearson correlations between anthocyanin content of pigmented pericarp samples and log_2_ TPM gene expression are illustrated

Correlational analysis of the top 10 DEGs of each genotype and anthocyanin content (µg/g DW) of pigmented pericarp tissue fractions detected highly expressed, influential candidate genes for anthocyanin production (Table 2). *Bz2* appeared within the top 10 DEGs of all pigmented genotypes. *Wrky33* appeared within the top 10 DEGs of B73 Color Converted, Mo17 Color Converted, and Amazonas; significant DGE of *Wrky33* was observed in Maize Morado outside of its top 10 DEGs. Specifically, three genes (*Bz2*, *Wrky33*, and *Aasr3*) showed the most extreme expression in Amazonas, which contains the highest concentration of pericarp anthocyanins (Fig. 9). *Bz2* has been implicated in anthocyanin transport/storage, supporting the role of transport/storage as a crucial mechanism for anthocyanin accumulation. *Aasr3* is in involved in abscisic acid hormone signaling and is activated during stress response (Liang et al. 2019; Rai et al. 2024). In maize aleurone, abscisic acid can activate expression of *C1*, the Myb factor of the MBW complex (Hattori et al. 1992). *C1* expression was absent from all samples suggesting abscisic acid may activate anthocyanin expression through interaction with the pericarp-specific Myb factor, *Pl1*. Upregulation of these 3 candidate genes is proposed to influence the higher anthocyanin content seen in Amazonas at 18 DAP (Table 2). While *Mrpa3* appeared in the 10 most highly expressed DEGs of Amazonas, it showed higher expression in one replicate of B73 Color Converted and is therefore not included in Fig. 9.

**Table 2:** The 10 highest DEGs per genotype were identified and correlated with anthocyanin content. A subset of these gene models with significant correlations to anthocyanin content are described below. Gene model corresponds to the Zm-B73-REFERENCE-NAM-5.0 reference genome

Gene model	Gene locus symbol	Correlation with anthocyanin content (*r*)	*P* value	Appearing in the highest 10 DEGs of pigmented genotypes
Zm00001eb048110	*Bz2*—*Bronze2*	0.86	3.52E − 04	Amazonas
				B73 Color Converted
				Maize Morado
				Mo17 Color Converted
Zm00001eb413920	*Wrky33*—*WRKY-transcription factor 33*	0.68	1.42E − 02	Amazonas
				B73 Color Converted
				Mo17 Color Converted
Zm00001eb376160	*Mrpa3*—*Multidrug resistance-associated protein3*	0.59	4.20E − 02	Amazonas
				B73 Color Converted
Zm00001eb083180	*Aasr3*—*Abscisic acid stress ripening3*	0.63	2.78E − 02	Mo17 Color Converted

#### Weighted correlation network analysis (WGCNA) of pigmented pericarp reveals two gene modules significantly correlated with anthocyanin content

A WGCNA grouped DEGs based on similar expression profiles into 10 gene modules. Two WGCNA gene modules were significantly correlated with pigmented pericarp anthocyanin content (µg/g DW): the positively correlated turquoise module (*r* = 0.65, *p* value = 0.023) and the negatively correlated pink module (*r* =  − 0.64, *p* value = 0.028). The turquoise and pink modules contained 6242 and 149 genes, respectively. In both modules, Amazonas, the genotype with the highest anthocyanin content, was found at one extreme end of the correlation. This genotype generally exhibited upregulation of genes in the turquoise module and downregulation of genes in the pink module.

**Fig. 10:**
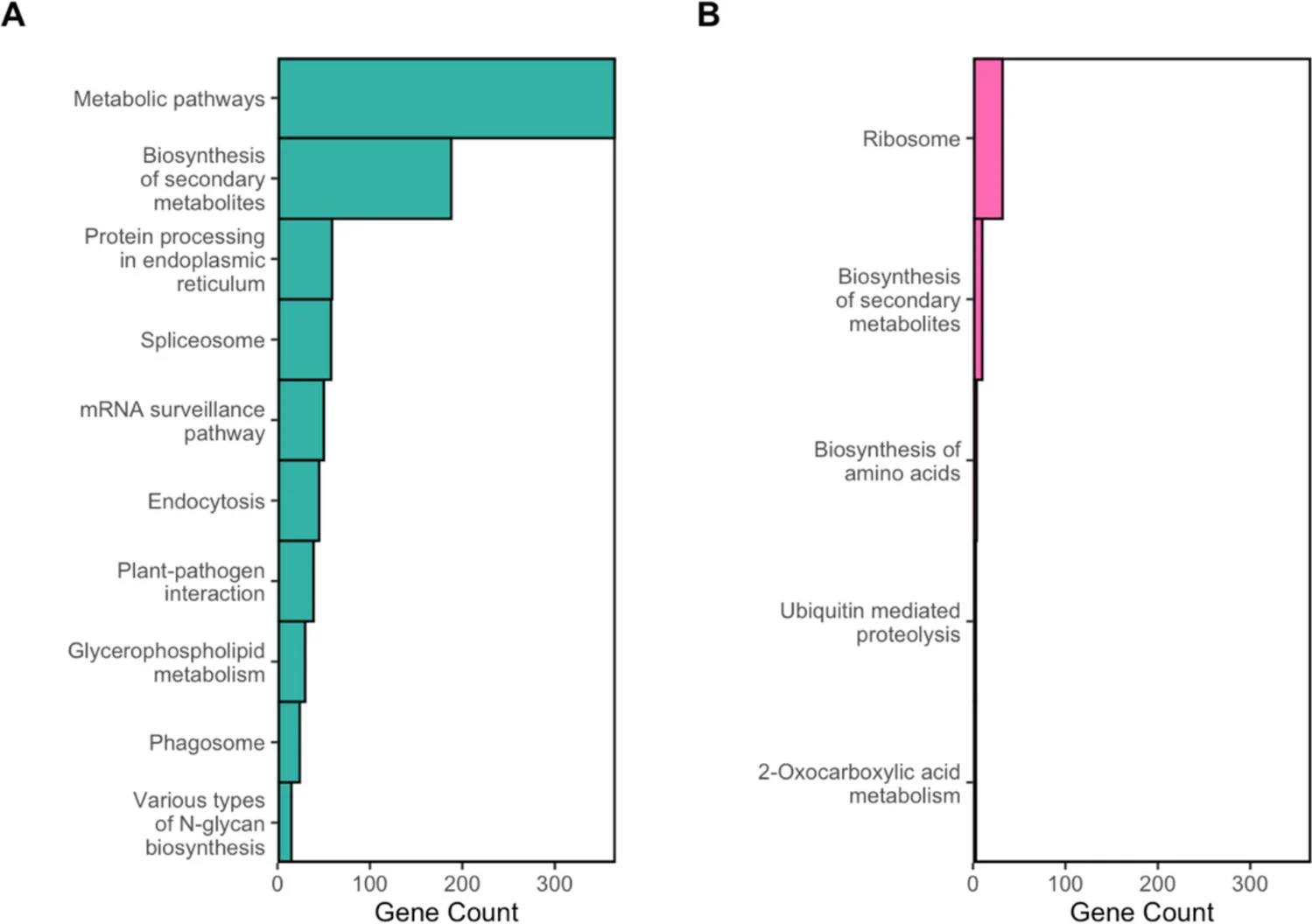
KEGG results of **(A)** the turquoise module and **(B)** the pink module. Significantly enriched pathways (FDR *p* ≤ 0.01) are displayed

The top 10 DEGs of the turquoise and pink modules, according to gene module membership, were used to define each module (Table 3). *WRKY-transcription factor 33* (*Wrky33*) was most strongly associated with the turquoise gene module (Table 3A). The remaining genes were implicated in stress response and signal transduction: [*NAC-transcription factor 5* (*Nactf5*), *molybdenum cofactor sulfurase1* (*Mocos1*), and *protein phosphatase homolog129* (*Prh129*)], growth and development [homologs of Arabidopsis FORMIN HOMOLOG 6 (Zm00001eb400670) and CYCLOPS 1 (Zm00001eb394460)], and transport (*Hak1*, Zm00001eb224870, Zm00001eb252970, Zm00001eb149250) (Luan 1998; Favery et al. 2004; Berardini et al. 2015; Heucken and Ivanov 2018; Watanabe et al. 2018; Qin et al. 2019; Kumar et al. 2021; Rajappa et al. 2024; Daniel-Mozo et al. 2024). Three known anthocyanin biosynthetic genes experienced high turquoise module membership (gene module membership > 0.6): *Bz2*, *Aat1*, and *Mrpa3* (Table S7). KEGG enrichment analysis of the turquoise module found most genes were associated with metabolic pathways and biosynthesis of secondary metabolites, consistent with high module membership of stress response and transport genes (Fig. 10A).

**Table 3:** Top 10 module defining genes of the turquoise and pink WGCNA modules ranked by gene module membership. Gene model corresponds to the Zm-B73-REFERENCE-NAM-5.0 reference genome. Gene trait significance represents the strength of association between gene expression and anthocyanin content. Gene module membership represents the strength of association between gene expression and the entire gene module. Significant genes were defined by high gene module membership and gene trait significance (gene module membership ≥ 0.6 and |gene trait significance|≥ 0.6) and DGE in at least one genotypic comparison (FDR *p* ≤ 0.01)

Gene model	Gene locus symbol	Gene module membership	Gene module membership *p* value	Gene trait significance	Gene trait significance *p* value
*(A) Turquoise*					
Zm00001eb413920	*Wrky33*—WRKY-transcription factor 33	0.978	3.53E − 08	0.684	1.42E − 02
Zm00001eb149250		0.977	4.92E − 08	0.651	2.17E − 02
Zm00001eb224870		0.975	6.92E − 08	0.700	1.13E − 02
Zm00001eb095110	*Nactf5*—NAC-transcription factor 5	0.973	1.04E − 07	0.626	2.93E − 02
Zm00001eb274240	*Mocos1*—molybdenum cofactor sulfurase1	0.972	1.26E − 07	0.702	1.09E − 02
Zm00001eb252970		0.972	1.26E − 07	0.664	1.86E − 02
Zm00001eb084630	*Hak1*—potassium high-affinity transporter1	0.971	1.43E − 07	0.624	3.00E − 02
Zm00001eb394460		0.968	2.34E − 07	0.647	2.30E − 02
Zm00001eb342010	*Prh129*—protein phosphatase homolog129	0.968	2.62E − 07	0.628	2.89E − 02
Zm00001eb400670		0.966	3.19E − 07	0.698	1.16E − 02
*(B) Pink*					
Zm00001eb379270		0.962	5.93E − 07	− 0.708	9.93E − 03
Zm00001eb079910		0.945	3.51E − 06	− 0.601	3.86E − 02
Zm00001eb291240		0.945	3.59E − 06	− 0.674	1.62E − 02
Zm00001eb354280		0.911	3.72E − 05	− 0.715	8.95E − 03
Zm00001eb321600		0.892	9.55E − 05	− 0.733	6.63E − 03
Zm00001eb275770	*Mctp9*—multiple C2 domain and transmembrane region protein9	0.883	1.40E − 04	− 0.602	3.82E − 02
Zm00001eb208240	*Fold3*—bifunctional protein FolD3	0.853	4.17E − 04	− 0.630	2.80E − 02
Zm00001eb254280		0.822	1.04E − 03	− 0.729	7.10E − 03

The pink module was negatively associated with anthocyanin content. Only eight DEGs met the criteria for gene module membership and gene trait significance, all of which were downregulated in pigmented pericarp (Table 3B). Specifically, the pink module was defined by genes related to growth and development (Zm00001eb254280, Zm00001eb291240, Zm00001eb321600, Zm00001eb354280, and Zm00001eb379270), intercellular signaling (*mctp9*), and folate metabolism (*fold3*) (Berardini et al. 2015; Brault et al. 2019; Zhang et al. 2021; Hawkins et al. 2025). No known anthocyanin biosynthetic genes were identified within the pink module. KEGG enrichment analysis of the pink module revealed most genes were associated with ribosomal functions (Fig. 10B). Although KEGG enrichment identified 10 genes involved in the biosynthesis of secondary metabolites, these genes primarily function in non-phenylpropanoid secondary metabolic pathways, such as carotenoid biosynthesis (data not shown). No direct phenylpropanoid precursor genes were found, suggesting the pink module genes may indirectly compete for energy and metabolic precursors.

## Discussion

To our knowledge, we have conducted the first temporal transcriptomic analysis of pericarp-pigmented corn within an elite field corn background. Visual inspection and UHPLC analysis of pigmented kernels from 10 to 20 DAP indicated anthocyanin accumulation over the course of kernel development (Fig. 3 and 4). Given that B73 Color Converted is a near-isogenic inbred line, its kernel developmental timings are more similar to B73 than Amazonas and Maize Morado. This is evident through the large number of DEGs at each timepoint of Amazonas and Maize Morado compared to B73 Color Converted. In contrast to the Amazonas and Maize Morado, B73 Color Converted showed a reduction in anthocyanin content (µg/kernel) at maturity compared to 20 DAP. It is unclear what metabolic processes are involved in anthocyanin degradation at maturity. Future studies will investigate the biochemical response associated with this reduction in anthocyanin concentration. Analyzing an additional timepoint between 20 DAP and kernel maturity may provide further insight into the processes controlling anthocyanin content during dry down in various pigmented genotypes.

In Experiment 1, analysis of the top DEGs identified the putative CHS gene, *C2*, and a pollen-specific CHS gene, *Whp1*, as consistently upregulated across all developmental timepoints of each pigmented genotype when compared to B73. While *Whp1* was originally characterized for its role in pollen pigmentation, recent detection in anthocyanin-rich maize vegetative tissues suggests its role extends beyond pollen-specific functions (Coe et al. 1981; Paulsmeyer and Juvik 2023b). However, analysis at 18 DAP revealed a lack of differential expression of CHS genes between pigmented and unpigmented pericarp of the same kernel indicating consistent expression throughout the entire pericarp tissue. CHS is an EBG that acts a shared precursor for all flavonoid biosynthetic pathways (Fig. 1A) (Dong & Lin 2021). Our data suggest early flavonoid precursors flux through various secondary metabolic pathways, including anthocyanin biosynthesis, indicating the gene products of *C2* and *Whp1* may contribute to multiple flavonoid pathways.

Overexpression of *Jac6* and *Ugt1*, whose roles have not been functionally characterized in anthocyanin biosynthesis, was seen in the top DEGs of all pigmented genotypes across kernel development, suggesting potential roles in enhancement of anthocyanin production. *Jac6* belongs to the jacalin-related lectin family, largely implicated in stress response, while *Ugt1* may act as an alternative UDP-glucosyltransferase to the putative *Bz1* (Dooner and Nelson 1977; Alagarasan 2024)*.* The jacalin-related lectin domain has previously been implicated in the interaction between *β*-Glucosidase-aggregating factor and *β*-Glucosidase, indicating a potential interaction between *Jac6* and *β-Glucosidase9* (*Glu9*), which is among the top 10 DEGs in B73 Color Converted and Maize Morado (Kittur et al. 2007). Strong upregulation of *Glu9*, a *β*-glucosidase enzyme with degradative, hydrolase activity, was found across the development of B73 Color Converted and Maize Morado (Ketudat Cairns and Esen 2010). Anthocyanin *β*-glucosidase (anthocyanase) is proposed to act in the degradation of anthocyanins to anthocyanidins in blood oranges (*Citrus* × *sinensis*), *Brunfelsia calycina* flowers, eggplant (*Solanum melongena*), and grapevine (*Vitis vinifera*) (Sakamura and Obata 1961; Barbagallo et al. 2007; Oren-Shamir 2009; Dong et al. 2019; Xie et al. 2021). Specifically, Barbagallo et al. 2007 noted an inverse relationship between increasing anthocyanase activity and anthocyanin concentration toward the end of blood orange fruit ripening. While the specific mechanism of anthocyanin degradation via anthocyanase is unknown, the pronounced expression of *Glu9* throughout the development of B73 Color Converted and Maize Morado, and the absence of expression in Amazonas, highlights a potential source of variation in anthocyanin concentrations between these different genotypes. Upregulation of *Ugt1* was consistent throughout all genotypes in Experiment 2, with the exception of Mo17 Color Converted (Table S5). Mo17 Color Converted lacks anthocyanin condensed forms (Fig. S2), highlighting *Ugt1* as a strong candidate enzyme for adding the 5-glucoside to the condensed form molecule. Additionally, a candidate MATE transporter, *Mate12*, was identified in the top 10 DEGs of B73 Color Converted, requiring further investigation into its specific role in flavonoid accumulation (Chatham and Juvik 2021).

When assessing the fixed loci that may be responsible for elevated anthocyanin concentration in the B73 Color Converted line when compared to B73, anthocyanin regulatory genes were identified. Anthocyanin content segregates 1:3 (purple: non-purple) in each backcross generation indicating that two genes are necessary for pericarp pigmentation (results not shown). Dense SNPs located on Chromosome 6 and Chromosome 10 implicate *Pl1* and *Lc1*, respectively, as the major contributors to anthocyanin content in the B73 Color Converted line due to the presence of fixed alleles within these loci (Fig. 6). The fixation of *P1* aligns with its characterized role in the activation of many phenylpropanoid pathway genes (Morohashi et al. 2012). Anthocyanin content was selected at each generation, which indirectly selected for *P1*. *P1* may be activating genes that shuttle more anthocyanin precursors into the pathway, increasing anthocyanin content. The concurrent activation of *Lc1*, *P1*, and *Pl1* explains how anthocyanin content was enriched in B73 Color Converted. The reason for the fixation of most of the distal arm of Chromosome 1 in B73 Color Converted is unclear (Fig. 6). Grain yield and adaptation to the Midwest were additional breeding goals for the B73 Color Converted line. The original purple parent was a photoperiod-sensitive landrace not very well suited for a Midwest climate. The final inbred was selected to perform similarly to B73 in terms of flowering time and grain yield. The fixed locus may be involved with grain yield, domestication traits, or flowering time and was selected indirectly. An alternative hypothesis is that the Chromosome 1 locus is associated with *Bz2.* It is possible that the purple landrace parent could have a more effective allele of this transport gene. Experiment 2 showed that *Bz2* expression was highly correlated with anthocyanin content (Fig. 9), emphasizing its role as a candidate for purple corn color enhancement.

While the MBW complex has been well-characterized in aleurone-pigmented maize kernels, the transcription factors involved in pericarp pigmentation were unknown. Along with being fixed in B73 Color Converted, it was demonstrated that *Pl1* and *Lc1* were significantly upregulated in all samples. It is known that some alleles of *B1* confer pericarp pigment (Chatham et al. 2019). The pericarp of the three intensely purple landraces chosen for this study, along with Apache Red from another study (Chatham and Juvik 2021), are all pigmented by *Lc1*, which suggests that *B1* is not a strong contributor to pericarp pigmentation due to its low expression. As for the WD40 member of the MBW complex, the two candidates in the maize genome are *Pac1* and *Mp1*. It was suggested in Paulsmeyer and Juvik (2023b) that *Mp1* may be the WD40 member for vegetative tissue. However, in the present study, expression of *Mp1* was lower than *Pac1* in pericarp tissue (10.23 vs. 82.95 TPM on average). This suggests that *Pac1* may be more important for activating anthocyanin synthesis in the grain overall. Many known anthocyanin repressor genes were also found to be upregulated in certain pericarp samples. If the goal is to make an anthocyanin-enriched variety, then *In1*, *Myb8*, *Myb31*, *Myb42*, and *Mybr97* expression should be reduced.

Flux through various flavonoid biosynthetic pathways was observed in addition to anthocyanin biosynthesis. It appears many precursors to anthocyanins were shuttled into making phlobaphenes, flavones, flavonol, and flavan-3-ols. Phlobaphenes must be present in mature samples since *P1* was active in all pigmented varieties except Mo17 Color Converted. While maysin, a flavone, has protective effects for the plant (Waiss et al. 1979; McMullen et al. 1998), it may be competing for potential anthocyanin precursors. The activation of the maysin pathway cannot be uncoupled from *P1*, which we have found to be important for increasing anthocyanin accumulation. Future breeding programs should be aimed at knocking down expression of *Fns1* to reduce phlobaphene and flavone synthesis and increase anthocyanin precursors. Similarly, *Fls1* and *Fls2* are in direct competition with DFR and produce colorless flavones (Choudhary and Pucker 2024). Reducing these compounds may help increase anthocyanin content. Flavan-3-ols are also colorless compounds that compete with anthocyanins, but these molecules are a component of condensed form dimers. The mechanism of condensed form anthocyanin formation is unknown, but it is theorized that *P1* is responsible for activating their synthesis (Chatham and Juvik 2021). In Mo17 Color Converted, *P1* expression and concordantly *Cgt1*, *Fns1*, *Sm1*, and *Sm2* expression were very low in pigmented pericarp tissues (Table S5). Condensed forms could not be detected in this line (Fig. S2), adding evidence that *P1* is involved in activating condensed forms. The upregulation of *Glu9* also indicates a potential method for anthocyanin degradation. This gene should be investigated for its function to determine whether it has a negative effect on anthocyanin accumulation.

Various correlational analyses of Experiment 2 identified two genes highly associated with enhanced anthocyanin accumulation: *Bz2* and *Wrky33* (Table 2 and 3, Table S7). While *Bz2* is known to be involved with vacuolar transportation (Marrs et al. 1995; Goodman et al. 2004)), the role of *Wrky33* in anthocyanin biosynthesis has yet to be characterized in maize. The closest homologs of *Wrky33* are *TRANSPARENT TESTA GLABRA 2* (TTG2) in Arabidopsis and *Ph3* in petunia (Gonzalez et al. 2016; Lloyd et al. 2017). TTG2 regulates proanthocyanidin biosynthesis through the enhanced expression of the vacuolar transport steps. Similarly, PH3, the WRKY33 homolog in Petunia, regulates vacuolar transport through the hyperacidification of anthocyanin-containing vacuoles via P-ATPase transmembrane transporters (de Vlaming et al. 1983; Tornielli et al. 2009; Faraco et al. 2014; Verweij et al. 2016). In addition to these genes, the LBGs, *Aat1*, *A2*, *Fht1*, *Fls2*, and *Mrpa3*, were activated in purple pericarp at 18 DAP. *Wrky33* appears to play a significant role in LBG activation similar to *TTG2* and *Ph3*. Both *Bz2* and *Wrky33* exhibited the highest DGE in Amazonas, the most abundantly pigmented genotype at 18 DAP (Fig. 9). Results of Experiment 2 show that LBG upregulation after 15 DAP is important for increasing anthocyanin content.

## Conclusion

We developed two near-isogenic purple pericarp inbreds (B73 Color Converted and Mo17 Color Converted) and two highly pigmented RILs in ex-PVP inbred backgrounds (Amazonas and Maize Morado). In Experiment 1, we conducted a temporal transcriptomic analysis of pericarp-pigmented maize lines vs. the reference inbred, B73. In this experiment, we found that the trio of regulatory genes, *Lc1*, *Pl1*, and *P1*, were found to be most important for activating anthocyanin biosynthetic genes in all pigmented lines. In addition, most of the anthocyanin biosynthetic genes previously identified in aleurone were also upregulated during pigmented pericarp development, despite differences in developmental origin (embryonic vs. maternal). Analysis of anthocyanin content during pericarp development identified a large increase in anthocyanin accumulation between 15 and 20 DAP in all pigmented genotypes, prompting further analysis of this critical timeframe in Experiment 2. In Experiment 2, we contrasted purple pericarp vs. non-purple pericarp of the same kernels using RNA-seq. Experiment 2 identified increased activation of LBGs in pigmented pericarp. Correlational and clustering analyses of DEGs found expression of transport-related genes, *Bz2*, *Mrpa3*, and *Wrky33*, that differentiated pigmented and unpigmented pericarp tissues. While *Bz2* and *Mrpa3* are known anthocyanin transporters, reports of *Wrky33* homologs in other species suggest it is a strong candidate gene for anthocyanin regulation. We identified upregulation of known anthocyanin repressors, *In1*, *Myb8*, *Myb31*, *Myb42*, and *Mybr97*, and a candidate anthocyanase, *Glu9*, in various pigmented samples. Together these loci are strong candidates for targeted selection through breeding—both to enhance anthocyanin concentrations and remove alleles associated with reduced pigmentation. Results from this study provide resources for purple corn breeders to increase anthocyanin content in maize cultivars as sources of natural supplements for processed foods and beverages. Future work will assay for the presence of anthocyanin degradation or anthocyanin complexed compounds using untargeted metabolomics and identify the allelic variation underlying enhanced anthocyanin production.

## Supplementary Information

Below is the link to the electronic supplementary material.

Supplementary file1 Micrograph images of mature kernel cross-sections of (A) B73 Color Converted, (B) Mo17 Color Converted, (C) Amazonas, and (D) Maize Morado. Images taken under 10x magnification using white light from above on an Olympus BX51 microscope (Olympus Corporation). Pericarp thickness was measured in the region opposite the scutellum. Pericarp thickness was positively correlated in anthocyanin content (r = 0.98) (DOCX 1179 KB)

Supplementary file2 UHPLC Chromatograms of 18 DAP pigmented pericarp tissue from (A) B73 Color Converted, (B) Mo17 Color Converted, (C) Amazonas, and (D) Maize Morado. Primary cyanidin-derived compounds are labeled: (1) Catechin-(4,8)-cyanidin 3,5-diglucoside, (2) Catechin-(4,8)-cyanidin 3-6″-malonylglucoside-5-glucoside, (3) Cyanidin 3-O-glucoside, (4) Cyanidin 3-6″-malonylglucoside, and (5) Cyanidin 3-3″,6″-dimalonylglucoside. Proportion of total peak area is attributable to cyanidin glycosides [Cyanidin 3-O-glucoside (3), Cyanidin 3-6″-malonylglucoside (4), and Cyanidin 3-3″,6″-dimalonylglucoside (5)]. Peak areas were measured in milli-Absorbance Units (mAU) (DOCX 4142 KB)

Supplementary file3 Alignment of expressed P1 duplications. High sequence similarity between the tandem repeats of the P1 locus interferes with transcript alignment (DOCX 758 KB)

Supplementary file4 The number of filtered SNPs in a 1 Mb window (DOCX 141 KB)

Supplementary file5 Summary of RNA-seq read alignments from Experiment 1. Table lists the number of input reads, uniquely mapped reads, and the percentage of uniquely mapped reads (XLSX 11 KB)

Supplementary file6 Summary of RNA-seq read alignments from Experiment 2. Table lists the number of input reads, uniquely mapped reads, and the percentage of uniquely mapped reads (XLSX 10 KB)

Supplementary file7 Log2 fold-change expression of genes depicted in Fig. 5A. Genes are divided into structural, regulatory, and transport/storage genes. Expression was measured as the difference in gene expression (TPM) between whole pericarp tissue of each pigmented genotype and B73 at 10, 15, and 20 DAP. FDR-adjusted p-values are reported (XLSX 19 KB)

Supplementary file8 (XLSX 6263 KB)

Supplementary file9 Log2 fold-change expression (TPM) between pigmented and unpigmented pericarp tissue of the same kernels at 18 DAP. FDR-adjusted p-values are reported (XLSX 4142 KB)

Supplementary file10 Log2 fold-change expression of genes depicted in Fig. 8A. Genes are divided into structural, regulatory, and transport/storage genes. Expression was measured as the difference in gene expression (TPM) between pigmented and unpigmented pericarp tissue of the same kernels at 18 DAP. FDR-adjusted p-values are reported (XLSX 14 KB)

Supplementary file11 All genes clustered within the turquoise and pink WGCNA modules ranked by gene module membership (XLSX 475 KB)

## Data Availability

Data supporting the findings of this work are available within the paper and its Supporting Information files. RNA-seq data are available in the Short Read Archive (SRA) database under the project identity PRJNA1235198 and PRJNA1235721 for Experiments 1 and 2, respectively. All other data generated and analyzed during the current study are available from the corresponding author upon request.
