# Supplementary figures and images for: Comparative transcriptomics of anthocyanin accumulation in the pericarp of pigmented purple corn
Source: Theor Appl Genet. 2026 Jan 17;139(1):37. doi: 10.1007/s00122-025-05137-x (PMC12812100; doi:10.1007/s00122-025-05137-x)

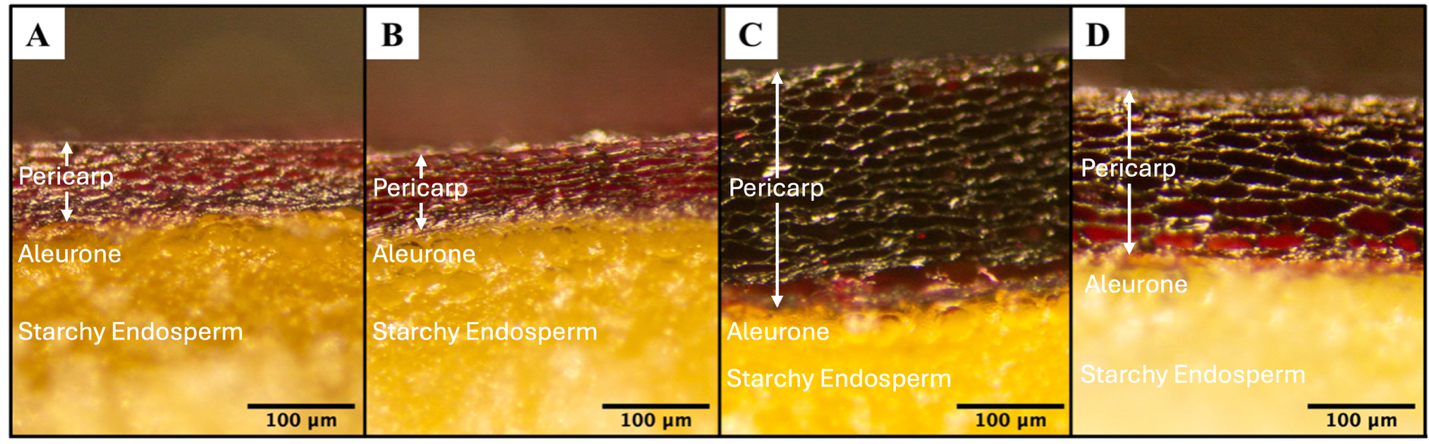

Supplement: Supplementary file 3 — Supplementary file3 Alignment of expressed P1 duplications. High sequence similarity between the tandem repeats of the P1 locus interferes with transcript alignment (DOCX 758 KB) [file 122_2025_5137_MOESM3_ESM.docx]

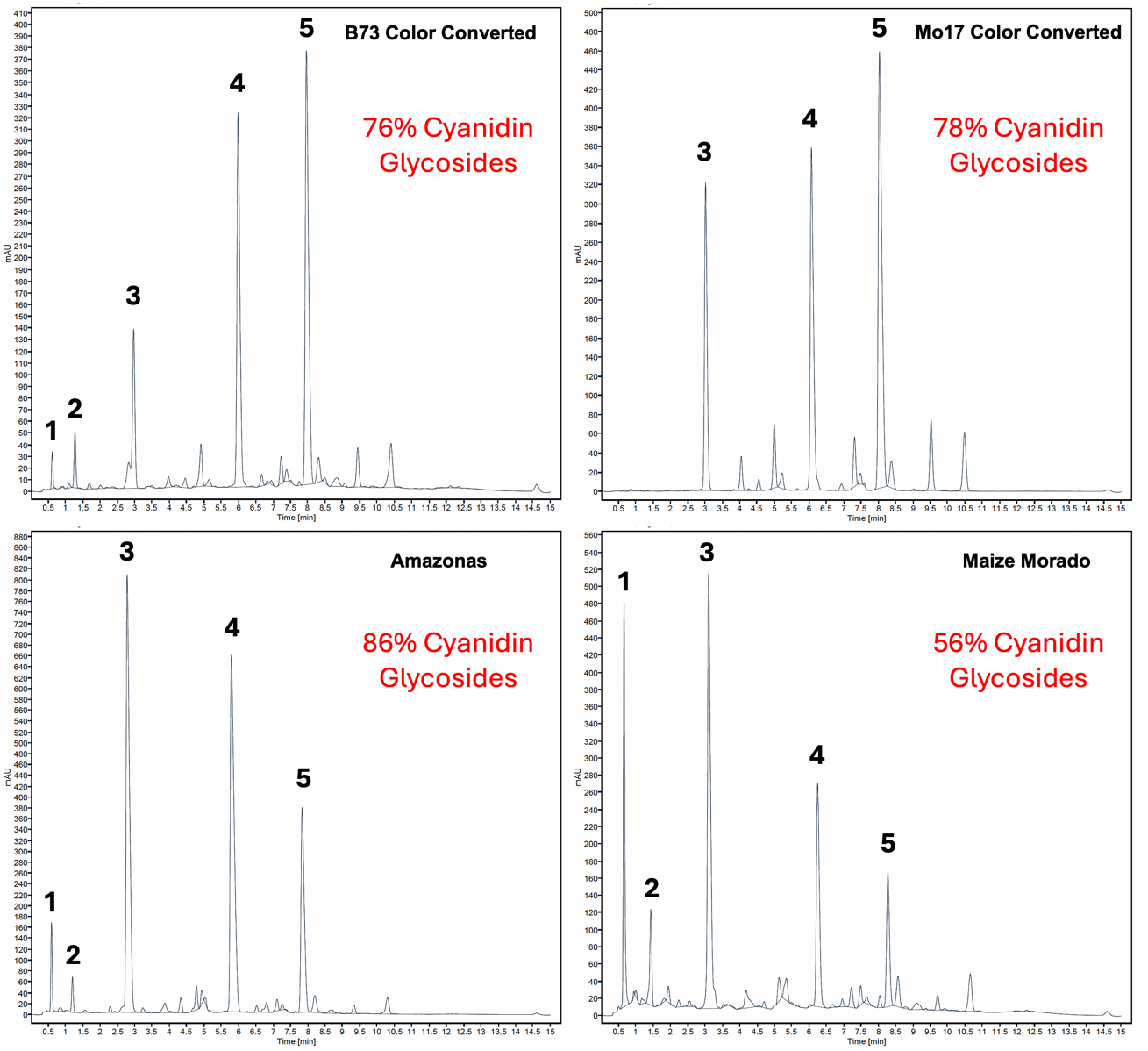

Supplement: Supplementary file 4 — Supplementary file4 The number of filtered SNPs in a 1 Mb window (DOCX 141 KB) [file 122_2025_5137_MOESM4_ESM.docx]
